# Targeting of Liver Mannan-Binding Lectin–Associated Serine Protease-3 with RNA Interference Ameliorates Disease in a Mouse Model of Rheumatoid Arthritis

**DOI:** 10.4049/immunohorizons.1800053

**Published:** 2018-09

**Authors:** Nirmal K. Banda, Dhruv Desai, Robert I. Scheinman, Rasmus Pihl, Hideharu Sekine, Teizo Fujita, Vibha Sharma, Annette G. Hansen, Peter Garred, Steffen Thiel, Anna Borodovsky, V. Michael Holers

**Affiliations:** * Division of Rheumatology, Department of Medicine, University of Colorado Anschutz Medical Campus, Aurora, CO 80045; † Alnylam Pharmaceuticals Inc., Boston, MA 02142; ‡ Skaggs School of Pharmacy, University of Colorado Anschutz Medical Campus, Aurora, CO 80045; § Department of Biomedicine, University of Aarhus, DK-8000 Aarhus, Denmark; ¶ Department of Immunology, Fukushima Medical University, Fukushima 960-1295, Japan; ∥ Division of Endocrinology, Metabolism and Diabetes, Department of Medicine, University of Colorado Anschutz Medical Campus, Aurora, CO 80045;; # Laboratory of Molecular Medicine, Department of Clinical Immunology, Section 7631, University Hospital of Copenhagen, 2200 Copenhagen, Denmark

## Abstract

Mannan-binding lectin–associated serine protease 3 (MASP-3) regulates the alternative pathway of complement and is predominantly synthesized in the liver. The role of liver-derived MASP-3 in the pathogenesis of rheumatoid arthritis (RA) is unknown. We hypothesized that liver-derived MASP-3 is essential for the development of joint damage and that targeted inhibition of MASP-3 in the liver can attenuate arthritis. We used MASP-3–specific small interfering RNAs (siRNAs) conjugated to N-acetylgalactosamine (GalNAc) to specifically target the liver via asialoglycoprotein receptors. Active GalNAc–MASP3–siRNA conjugates were identified, and in vivo silencing of liver MASP-3 mRNA was demonstrated in healthy mice. The s.c. treatment with GalNAc–MASP-3–siRNAs specifically decreased the expression of MASP-3 in the liver and the level of MASP-3 protein in circulation of mice without affecting the levels of the other spliced products. In mice with collagen Ab–induced arthritis, s.c. administration of GalNAc–MASP-3–siRNA decreased the clinical disease activity score to 50% of controls, with decrease in histopathology scores and MASP-3 deposition. To confirm the ability to perform MASP-3 gene silencing in human cells, we generated a lentivirus expressing a short hairpin RNA specific for human MASP-3 mRNA. This procedure not only eliminated the short-term (at day 15) expression of MASP-3 in HepG2 and T98G cell lines but also diminished the long-term (at day 60) synthesis of MASP-3 protein in T98G cells. Our study demonstrates that isoform-specific silencing of MASP-3 in vivo modifies disease activity in a mouse model of RA and suggests that liver-directed MASP3 silencing may be a therapeutic approach in human RA.

## INTRODUCTION

Rheumatoid arthritis (RA), a crippling inflammatory autoimmune disease of the joints, affects ~0.24% of the world population (1). The severe morbidity of this condition indicates that this disease will impact the public health care system dramatically (2). Although the advent of biologics for the treatment of RA has improved outcomes, ~40% of patients respond partially or not at all. Despite clinical improvement afforded by these biologic and small molecule therapeutics, the radiological damage in RA can continue over time because of the underlying inflammatory processes that are not fully controlled by the existing drugs on the market (3).

We and others have extensively probed the complement network to identify targets suitable for therapeutic development (4–7). Because of the complexity of human RA, current drug targets include cytokines (TNF-α, IL-1, and IL-6) as well as cells (B cells and T cells). We suggest that complement may serve as an additional important therapeutic target to add to the RA armamentarium, whichalsomayconferbenefit becausecomplementactivationexerts its function upstream in the inflammatory cascade.

The etiology of RA is complex and involves a combination of environmental, hormonal, genetic, and epigenetic elements leading to an increased likelihood of developing an immune response to posttranslationally altered peptides. Citrullinated peptides are considered a prime example, and the presence of anticitrullinated peptide Abs (ACPAs) is both disease-specific and significantly correlated with radiological damage in patients with RA (3). However, the development of autoantibodies to altered self alone is not sufficient to trigger RA. Rather, a second hit has been postulated to be required for localization of this immune response to the joint (8). Ultimately, it appears that the deposition of Ab within the synovial space leads to the initiation of a joint-specific immune response, which becomes self-sustaining. Our hypothesis is that the liver plays a critical role in this second hit process through the secretion of potent proinflammatory complement proteins.

The complement system is composed of many soluble proteins and receptors, a substantial proportion of which are produced by the liver (9). Other tissues, however, can secrete complement components, including epithelial, endothelial, and immune cells (10, 11). The complement system is activated through three different pathways: the classical pathway, the lectin pathway (LP), and the alternative pathway (AP). All of these pathways converge with the cleavage of C3 and C5 via C3 and C5 convertases, respectively, generating C3a, C3b, C5a, and C5b. C3a and C5a function as general activators of inflammatory cells via C3a and C5a receptors, whereas C5b promotes the assembly of the membrane attack complex C5b-9.

It iswell accepted basedon studies ofcomplement component-deficient strains in mouse models of arthritis that this system plays an essential role in the development of experimental models of RA (12–16). Moreover, activated complement fragments have been found in the synovium of RA patients (15, 17, 18). Additionally, two mouse models of arthritis (i.e., collagen-induced arthritis and collagen Ab–induced arthritis [CAIA]) have extensively been used to successfully test many drugs currently being used for the treatment of RA (19, 20).

The LP of the complement system is activated when pattern recognition molecules (PRMs) binds to targets. These PRMs include mannan-binding lectin (MBL), collectin-L/K (collectin-10/11), H-ficolin (ficolin-3), L-ficolin (ficolin-2), or M-ficolin (ficolin-1) (21). Associated with the PRMs are a set of proteases that link PRM binding to activation of the complement system. To this end, the liver synthesizes three different proteases of the LP that play this role: MBL-associated serine protease (MASP)-1, MASP-2, and MASP-3 and two MBL-associated proteins, sMAp (MAp19 or MAP-2) and MAp44 (MAP-1) (22–25). The *MASP1* gene generates via alternative splicing the two proteases MASP-1 and MASP-3 as well as MAp44 (26, 27), whereas the MASP2 gene generates MASP-2 and sMAp (25).

WhenPRMs bind to relevant patterns, MASP-1 is activated and then promotes the activation of MASP-2, which subsequently cleaves C4 and C2, thereby generating the C3 convertase, C4b2a (28). In vitro (not in vivo) studies have shown that MASP-1 is essential for bacterial LPS but not zymosan-induced AP (29), indicating that MASP-1 can regulate a specific AP subset and not the entire AP. MASP-3 is required for the cleavage of profactor D (proFD) into mature factor D (FD), an essential factor of the AP and amplification loop, which is engaged and boosts the activity of all pathways of the complement system (22, 28, 30–33). Mice lacking MASP-1/3 are strongly resistant to CAIA (14), whereas mice lacking MASP-2 are partially resistant (34). The individual roles of MASP-1 and MASP-3 could not be resolved in these studies, as the knockout of the *MASP1* gene eliminates both splice variants. In addition, *MASP-1/3*^*−/−*^ exhibit developmental delays and small size. Both *MASP-1/3*^*−/−*^ mice lacking the enzymes as well as wild type (WT) mice treated with a mixture of commercial MASP-1/3 small interfering RNAs (siRNAs) were protected from the CAIA (22). Because of the interrelationships of the systems, the individual role of MASP-1 or MASP-2 or MASP-3, however, remained to be determined.

RNA interference (RNAi) is a naturally occurring silencing mechanism that has been used to develop a highly efficient and targeted technology to silence pathogenic genes using synthetic siRNAs or intracellularly generated siRNAs using short hairpin (sh)RNAs. In this study, we used two different RNAi delivery approaches to silence the MASP-3 transcript specifically in cells and in mouse liver. We used lentiviral constructs encoding shRNAs targeting MASP-3 for efficient and long-term silencing of MASP-3 in human cell lines (35).

The asialoglycoprotein receptor (ASGPR) is highly expressed on hepatocytes and binds triantennary *N*-acetylgalactosamine (GalNAc) ligands with nanomolar affinity (36, 37). The ASGPR is conserved across species, is highly expressed on hepatocytes (~500 K/cell) (38–40), and has a 15-min recycling time following internalization (41). These properties of the ASGPR facilitate rapid and specific uptake of GalNAc-siRNAsconjugatesintohepatocytes (reviewed in Ref. 42). The GalNAc-siRNA conjugate technology has been used to target multiple liver-expressed genes and has demonstrated robust silencing in preclinical animal models and clinical studies. As an example, 90% knockdown was obtained of the liver-expressed transthyretin gene in a phase I study of the GalNAc-siRNA ALN-TTRsc (reviewed in Ref. 42). More recently, it has been shown that a single dose of Inclisiran (ALN-PCSsc), a GalNAc-conjugated siRNA targeting proprotein convertase subtilisin-kexin type 9 (PCSK9) robustly reduced serum PCSK9 levels, resulting in the lowering of a target for the lowering of low-density lipoprotein cholesterol for up to 6 mo after a single dose (43). These studies provide direct clinical proof of concept that GalNAc-conjugated siRNAi are a good choice for hepatocyte-targeted delivery of GalNAc–MASP-3–siRNA duplexes to silence liver-derivedMASP-3expressionandthustospecificallyinvestigate the role of MASP-3 in inflammatory arthritis.

Our first objective in this study was to demonstrate that human shMASP-3 (hu shMASP-3) RNA can be used to silence MASP-3 in human cells expressing high levels of MASP-3. Our second objective in this study was to generate a GalNAc–MASP-3–siRNA duplex targetingthe liver andto test its efficacy in vivo in mice with and without arthritis. Our hypothesis is that liver-derived MASP-3 is essential for the development of joint damage and that its targeted hepatic inhibition can lead to the attenuation of arthritis.

## MATERIALS AND METHODS

### Determining the expression of MASP-3 in human cell lines and in mouse and human liver

The human glioblastoma multiforme brain tumor cell line T98G and the human liver carcinoma cell line HepG2 were grown in Eagle’s MEM (Invitrogen by Thermo Fisher Scientific, Carlsbad, CA) containing 5% FBS (HyClone, Logan, UT) and maintained at 37°C in 5% CO_2_ (44). Liver from WT C57BL/6 mouse was dissected, washed in 1× PBS, and used to make the RNA. Total RNA from the normal human liver was obtained from outside sources as a residual material. Total RNA was extracted from the human cell lines or from the mouse liver using the Qiagen RNeasy Kit. RNA quality was validated prior to use by agarose gel electrophoresis. Human or mouse MASP-1, MASP-2, and MASP-3 expression in cells and in liver were determined by quantitative RT-PCR (qRT-PCR) as described previously (5, 45). Primer sequences used for amplification of human or mouse MASP-1–, MASP-2–, and MASP-3–encoding RNA are available on request from the authors. Standard curves to calculate the expression of MASP-1, MASP-2, and MASP-3 were made using RNA from the HepG2, T98G cell lines, WT mouse liver, and from the human liver.

### RT-PCR for MASP-1, MASP-2, and MASP-3 expression in cells and in liver

T98G or HepG2 cells or mouse liver tissues were lysed, and total RNA was prepared using RNeasy Mini Kit (Qiagen, Germantown, MD). Absence of RNA degradation was assessed by using a 1% agarose gel, and cDNA was prepared (5, 45). MASP-1, MASP-2, and MASP-3 mRNA were measured by RT-PCR. Quantitation of 18S rRNA (18S rRNA) was used as an internal control in each experiment, and all data were expressed in picograms per nanogram mRNA/18S rRNA. All mRNA samples were analyzed in duplicate. Samples were analyzed by amplifying at 40 cycles according to the methods published previously in Nature Protocols (5, 45). All qRT-PCR data were analyzed by using cDNA based on the standard curve made by using liver RNA from a healthy human subject or normal WT mice.

### Generating hu shMASP-3 RNA and shScramble RNA vesicular stomatitis virus lentivirus particles and transduction of human cells

hu shMASP-3 RNA targeting human MASP-3 (NM_139125.3) (mission TRCN0000429394; Sigma-Aldrich, St. Louis, MO) and shScramble RNA (mission pLKO.1-CMV-puro, SHC002; Sigma-Aldrich) were obtained from the Functional Genomic Core of the University of Colorado at Denver.

hu shScramble RNA was used as a negative control in each experiment. The human embryonic kidney 293 cells transformed with the SV40 large T Ag (HEK293T) packaging cell line was used to package hu shMASP-3 RNA and to generate lentivirus viral particles. Briefly, 2 μg of shMASP-3 plasmid was combined with 2 μg of packaging vector (3:1 M ratio of pΔ8.9 and pCMV-VSV-G at 100 ng/μl), diluted in 400 μl of Opti-MEM (31985070; Life Technologies), and incubated with 12 μl of 1 mg/ml polyethylenimine (Polysciences, Warrington, PA) for 15–20 min at room temperature. The entire contents of the tube were added to HEK293FT cells and incubated overnight. After transfection for 16 h, the media on the cells were replaced with complete DMEM. Forty-eight hours later, the media containing viral particles were collected from transfected HEK293FT cells, transferred to a 5-ml syringe, and filtered through a 0.45-μM cellulose acetate filter (VWR, Radnor, PA), and used immediately.

Transduction efficiency was established at various time points using GFP-expressing viral particles, TurboGFP (SHC003; Sigma-Aldrich). A total of 3 × 10^6^ T98G or HepG2 cells per well (12-well plate) were infected with 1.6 × 10^3^ infectious units (16-μl volume) of lentivirus particles expressing either shMASP-3 RNA or shScramble RNA. After 24 h, viral media were replaced with media containing puromycin (10 μg/ml).

To explore the short-term (at day 15) or the long-term (at day 60) effect of hu shMASP-3 RNA on MASP-3 silencing, the following in vitro experiments were conducted. In the first experiment to examine the expression of MASP-3 in T98G or HepG2 cells, after transfection with shMASP-3 RNA or shScramble RNA, cells were harvested and lysed to make the total RNA at day 15. To further explore the extended long-term effect of hu shMASP-3 RNA or of hu shScramble RNA on MASP-3 protein at day 60, MASP-3 expression in T98G cell lysates was assessed by qRT-PCR. We have not used HepG2 cells to show the longer-term effect at day 60 because of the longer-term toxicity of hu shMASP-3 in this particular cell line.

### Source of human and mouse serum lacking MASP-1/3 or MASP-2

Normal human serum and FD-depleted sera were obtained from Complement Technology, Tyler, TX. Human and mouse serum samples were stored at 270°C and were thawed only one time prior to use. Human serum deficient in MASP-2 and MASP-1/3 for these studies were kindly provided by Dr. P. Garred (Copenhagen, Demark) and Dr. S. Thiel (Aarhus, Denmark), respectively. We have established a colony of *FD*^*−/−*^, *MASP-1/3*^*−/−*^, and *MASP-2*^*−/−*^ miceonC57BL/6background.Sera fromWT,*FD*^*−/−*^,*MASP-1/3*^*−/−*^, and *MASP-2*^*−/−*^ mice were obtained through retro-orbital bleeding according to the approved protocol by the Institutional Animal Care and Use Committee. All mice used in this study were on C57BL/6 background. WT C57BL/6 mice were also purchased from The Jackson Laboratory to obtain serum for various studies. All mice were kept in our barrier facility and fed breeder chow provided by the Center for Laboratory Animal Care, University of Colorado Anschutz Medical Campus.

### Analysis for ProFD and FD of mouse and human serum deficient in MASP-1/3 and MASP-2 proteins

The presence or absence of ProFD and FD was detected by Western blotting of mouse and human serum deficient in either MASP-1/3 or MASP-2. Sera from *FD*^*−/−*^ mice and also human sera depleted in FD were also examined, which served as negative controls. Normal sera from WT C57BL/6 mice and normal human sera were used as positive controls in all experiments. Before Western blotting, human serum (25 μl) was preincubated with affinity-purified goat anti-human FD (2 μl) (R&D Systems, Minneapolis, MN), allowing ProFD and FD to be immunoprecipitated with Protein A/G beads (12 μl) (Santa Cruz Biotechnology, Dallas, TX). The immunoprecipitates were then treated with peptide-N-glycosidase F (PNGase F) overnight at 37°C according to the manufacturer’s suggested protocol (New England BioLabs, Ipswich, MA). The samples were analyzed by SDS-PAGE and Western blot. After transfer, the blots were incubated for 1 h with biotinylated anti-human anti-FD Ab as an alternative Ab that binds to a different epitope than the primary anti-human FD Ab used for precipitation (dilution 1:500) (R&D Systems). Finally, the proFD and FD bands at ~26 kDa were detected using streptavidin HRP conjugate (dilution 1:1000) (R&D Systems) and luminescent substrate. The band of proFD was visually slightly bigger in kDa than the FD band, and these differences were only visible by using 12% Bis-Tris Gel (14, 22, 28).

### In vivo AP activation in MASP-1/3^−/−^ mice by recombinant MASP-3

To examine the cleavage of ProFD by rMASP-3 in vivo in *MASP-1/3*^*−/−*^ mice, (male and female) were injected only oncei.v. in the tail with active or inactive rMASP-3 (serine changed to alanine) (50 μg/mouse). After 24 h of injection with rMASP-3 protein, sera from all *MASP-1/3*^*−/−*^ (n = 4) male and female mice were examined ex vivo for AP activation. The AP activation in serum at various dilutions (1:6 and 1:12 in EGTA/Mg buffer) was determined by rabbit erythrocyte lysis analysis as described earlier (32). Serum from a WT control mice was used as a positive control at various dilutions (1:6, 1:12, 1:24, and 1:48 in EGTA/Mg buffer) in duplicate to show the specificity of rabbit erythrocyte lysis for AP activation. The absorbance was read at 405 nm.

The serum samples before and after treatments with rMASP-3 were also analyzed for the cleavage of proFD into mature FD using Western blot analysis.

### In vitro selection of active GalNAc–siRNA–MASP-3 duplexes

Fifty mouse siRNA sequencesweredesigned based onthe available bioinformatics information to target the MASP3 splice variant of the *MASP1* gene. GalNAc-siRNA conjugates were synthesized using solid-phase synthesis methods as previously described (46). Cos-7 cell line was used to examine the effect of MASP-3 siRNA duplexes on the expression of MASP-3 in vitro. A customized fusion psiCHECK vector (Blue Heron Biotech, Bothell, WA) of luciferase reporter gene and mouse MASP3 was diluted with Opti-MEM for a final concentration of 1.2 ng/ml. MASP-3–siRNA and control siRNAs were diluted eight times at a 6-fold serial dilution beginning at 10 nM. The siRNA dilutions were cotransfected with the psiCHECK vector (1:1 ratio) and preincubated for 15 min with Lipofectamine (1:10 ratio)(Invitrogen by Thermo Fisher Scientific, Waltham, MA). The cell suspension was added to each well with the lipid/siRNA mix and incubated for 48 h at 37°C in an atmosphere of 5% CO2. Dual-Glo Reagent (Promega, Madison, WI) was used as the luminescence according to the manufacturer’s protocol and measured using Biotek Synergy HT plate reader. Each data point was tested with at least four biological replicates. Renilla luciferase reporter was used as the positive control. The magnitude of siRNA activity was then normalized relative to the negative control (psiCHECK2-MASP3 transfected but either nontargeting siRNA or PBS). All transfections were done at *n* = 2 or greater.

### psiCHECK2 Dual-Glo luciferase in vitro assay for MASP-3 gene silencing

The psiCHECK2 Dual-Glo system enables detection of siRNA-mediated silencing of target sequences fused to a Renilla luciferase reporter. The sequence of interest (MASP-3) was cloned into the multiple cloning region (XhoI-NotI sites) located downstream of the Renilla STOP codon in the 3’UTR. RNAi-mediated cleavage and degradation of the fusion mRNA can be measured by a loss in Renilla signal following siRNA treatment. The psiCHECK2 vector also contains a second reporter, firefly luciferase, which is driven by a different promoter and allows for normalization of Renilla expression. When MASP-3 siRNAs are transfected in Cos-7 cells followed by silencing of MASP3 gene, the levels of luciferase signal also go down. IC_50_ of each MASP-3 duplex was calculated from the expression curve. The IC_50_ value has been listed for each MASP-3 duplex on the plots. For example, duplex 4 (IC_50_ = 1.2 pM) is the more effective, as lower concentration of the duplex is enough to silenceMASP-3expressionby50%, whereasduplex1(IC_50_=41.2pM) is less effective in comparison.

### In vivo selection of active GalNAc–MASP-3–siRNA duplexes

The effect of MASP-3 siRNA by duplex 1 (duplex 3) and duplex 2 (duplex 4) was initially characterized in 8-wk-old naive C57BL/6J mice (Charles River Laboratories), and later on, these duplexes were further chemically modified. Single 1 or 10 mg/kg doses of duplex 1 and duplex 2 were administered s.c. Livers were collected on day 7 postinjection. The mRNA was isolated using RNeasy Plus Mini Kit (Qiagen). High-Capacity cDNA Reverse Transcription Kit (Thermo Fisher Scientific) was used to convert the mRNA samples. Levels of MASP3 expression were analyzed using qRT-PCR reagents compatible with Roche Lightcycler 480. The MASP3 mRNA was measured using a customized TaqMan assay (Thermo Fisher Scientific) and normalized to the endogenous GAPDH expression (Thermo Fisher Scientific).

In another experiment, C57BL/6J mice were injected with duplex 1 or with duplex 2 on day 25, day 0, and day 3 followed by liver collection at day 10, as this would be a relevant time point in a CAIA model (see below). Serum was obtained prior to injections to examine the levels of circulating MASP-3 by Western blot and ELISA. Liver was collected on day 10 and examined for MASP-3 expression using qRT-PCR.

Lastly, WT mice (*n* = 10) were injected with GalNAc–luciferase–siRNA or with duplex 2 of GalNAc–MASP-3–siRNA on day 210, day 0, and day 3. Sera from mice were obtained prior to injections to examine the circulating levels of MASP-3 protein by Western blot and ELISA. Liver was collected on day 10 and tested for MASP-3 mRNA expression using qRT-PCR. The impact of GalNAc–MASP-3–siRNA duplexes on MASP-1 levels was tested by examining the serum for MASP-1 levels by ELISA.

### Western blot analysis for human MASP-3 in T98G cell supernatants following MASP-3–encoding mRNA knockdown by shRNA and for MASP-1 and MASP-3 from mouse serum following siRNA treatment

Infected and controlT98G cell culture supernatants at day 60were collected and concentrated by centrifuging in Centricon Plus-70 Filter Units at 3000 rpm for 30 min (Millipore, Bedford, MA). MBL/MASP-1 or MBL/MASP-3 complexes were pulled out by incubating 20 μl of concentrated supernatant with 20 μl of mannan-agarose (Sigma-Aldrich) at 4°C overnight. The beads were washed and then subjected to 4–12% Tris-Glycine Gel (Invitrogen by Thermo Fisher Scientific) SDS-PAGE. Similarly, to analyze the effect of GalNAc–luciferase–siRNA or GalNAc–MASP-3–siRNA on MASP-3 in vivo, 20 μl of serum from each mouse was incubated with mannan-agarose as described above. All samples were transferredto 0.2 μm of microporous Immobilon P^SQ^ Transfer Membrane, polyvinylidene difluoride (PVDF) membrane (7 cm × 8.4 cm size) (Merck Millipore, Tullagreen, Carrigtwohill, County Cork, Ireland) using Tris-Glycine Transfer Buffer (Invitrogen by Thermo Fisher Scientific) for 1 h at 100 V. The PVDF membrane was blocked in 5% dry milk in 1× PBS containing 0.05% Tween 20 for 1.5 h and incubated overnight at 4°C with either 1) human anti–MASP-3 primary Ab (1:200 dilution) (Santa Cruz Biotechnology) 5% dry milk for analysis of human MASP-3 derived from T98G cells or 2) rabbit anti–MASP-3 primary Ab at a 1:200 dilution (produced in-house at Dr. Thiel’s laboratory) in 5% drymilk for analysis of MASP-3in mouseserum. All blots were washed 3 times in 1× PBS containing 0.5% Tween 20 then incubated with either 1) HRP-conjugated goat anti-mouse (1:5000 dilution) (Santa Cruz Biotechnology) or 2) HRP-conjugated goat anti-rabbit (1:5000) (Jackson Immuno-Research, West Grove, PA) diluted in 5% milk containing 0.5% Tween 20.

Finally, the membranes were developed from 1 to 5 min using a 1:1 mixture of SuperSignal West Pico Chemiluminescent Substrate (Thermo FisherScientific). Different concentrationsofa truncated form of the recombinant human MASP-3 protein were also used as a positive control along with normal human serum. The blots were scanned using GeneGnome XRQ Chemiluminescence Imaging SystemandGeneTools analysis software fromSyngene(Frederick, MD), and the density of each band was quantified using the Quantity One software (Bio-Rad, Hercules, CA).

The procedure for measuring MASP-1 was identical to that described above. The PVDF membrane was blocked and then probed with rabbit anti–MASP-1 primary Ab (1:200) in 5% dry milk. Sera from WT and *MASP-1/3*^*−/−*^ mice were used as positive and negative controls, respectively.

### Assay to measure MASP-3 protein by time-resolved immunofluorometric immunoassay in the circulation

Serum samples from various cohorts from WT C57BL/6 mice with no disease or with CAIA injected s.c. with GalNAc–luciferase–siRNA or with duplex 1 or with duplex 2 of GalNAc–MASP-3–siRNA were analyzed by time-resolved immunofluorometric immunoassays as previously described (26, 32, 47) by using in-house Abs. Sera were diluted 1:50 to measure the levels of MASP-3 in mouse serum. For MASP-3, sera from WT and *MASP-1/3*^*−/−*^ mice were used as positive and negative controls, respectively.

### Effect of GalNAc–MASP-3–siRNA on induction of CAIA in mice

WT mice were injected s.c. with 10 mg/kg on days 210, −5, and on day 0 either with GalNAc–luciferase–siRNA (*n* = 22) or GalNAc–MASP-3–siRNA (duplex 2) (*n* = 22). Then, CAIA was induced in WT mice by using a mixture of five mAb to bovine collagen type II (Arthrogen-CIA, Chondrex) resuspended in sterile Dulbecco’s PBS according to our previously published studies (13, 15, 34, 48). All WT mice received i.p. injections of 8 mg of Arthrogen on day 0 and 50 mg of LPS from Escherichia coli strain 0111B4 on day 3. All mice were sacrificed at day 10 post–LPS injection. The total duration of the CAIA experiment was day 20 after the first siRNA injection. The severity of clinical disease activity (CDA) in all groups of WT mice was determined every day by two trained laboratory personnel acting independently and blindly according to our previously published study (4). The in vivo CAIA experiments were repeated three times (total *n* = 44) using an identical dose of duplexes and an identical injection schedule.

### Assessment of histopathology, C3 deposition, and macrophage in joints

At day 10, both forepaws and the entire right hind limb, including the paw, ankle, and knee were surgically removed and fixed immediately in 10% buffered formalin (Biochemical Sciences). The preparation of knee joint samples and histological analysis using toluidine blue (T blue) dye were performed as previously described (14, 15). All sections were read by a trained observer who was also blinded to the treatment and to the disease activity score of each mouse. The joint sections from mice were scored for the changes in inflammation, pannus, cartilage damage, and bone damage on a scale of 0–5. C3 deposition in the joints (synovium and cartilage) was localized with polyclonal goat anti-mouse C3 antisera (ICN Pharmaceuticals) and scored as previously described (13). Macrophages infiltration in the knee joints were localized with rat anti-mouse F4/80 Ab (Bio-Rad) and scored using a scale from 0 to 4.

### Immunohistochemical localization of MASP-3 protein in the joints of mice

All joints from one CAIA experiment (*n* = 14) used for histopathology studies after treatments with GalNAc–luciferase–siRNA or GalNAc–MASP-3–siRNA described above were collected for further immunohistochemical (IHC) localization of MASP-3 protein. All five joints (i.e., both forelimbs and the right hind limb) (knee joint, ankle, and hind paw) were processed for IHC studies and for the MASP-3 deposition in the joints. Liverand brain from WT were used as positive controls, whereas liver from *MASP-1/3*^*−/−*^ mice was used as negative control. Five micron–thick paraffin sections were prepared for immunodetection and stained with rabbit anti-mouse MASP-3 biotinylated Ab at a dilution of 1:200. Sections required modest retrieval in 10 mM sodium citrate pH 6 for 10 min at 110°C in the NxGen Decloaker (BiocareMedical,Concord,CA) with a 10min cool down. MASP-3 immunodetection was performed on the Benchmark XT autos-tainer (Ventana Medical Systems, Tucson, AZ) at an operating temperature of 37°C with primary incubation for 32 min using a modified iVIEW DAB (Ventana Medical Systems) Detection Kit. The iVIEW secondary Ab was replaced with full-strength Rabbit ImmPRESS polymer (Vector Laboratories, Carpinteria, CA), and the enzyme was replaced with diluted Rabbit ImmPRESS polymer (Vector; 50% v/v in PBS pH 7.6). All sections were counterstained in hematoxylin for 2 min, blued in 1% ammonium hydroxide, dehydrated in graded alcohols, cleared in xylene, and cover glass mounted using synthetic resin. Negative controls to confirm the specificity of the immunostaining included omission of the primary Ab incubation step in the IHC protocol, substitution of the primary Ab diluent. Because of the overall low deposition of MASP-3 in the joints, an ordinal scale was used to assess levels.

### Statistics

All data were analyzed using GraphPad Prism 4.0 program. The CDA data from all individual WT mice were included in the final analysis of histolopathology and immunohisto-chemistry. The density of bands from Western blots was determined by using Bio-Rad Quantity One program after scanning and subtracting the background from blots. To analyze mRNA data from T98G or HepG2 cells and in mouse and human liver, t test was used. The test of ANOVA was used to find the statistical differences comparing more than three independent treatment groups. All data were expressed as the mean ± SEM with *p* < 0.05 considered significant, and original *p* values have been shown.

## RESULTS

### Expression of MASPs in different human cell lines, in mouse, and in human liver

**FIGURE 1.**
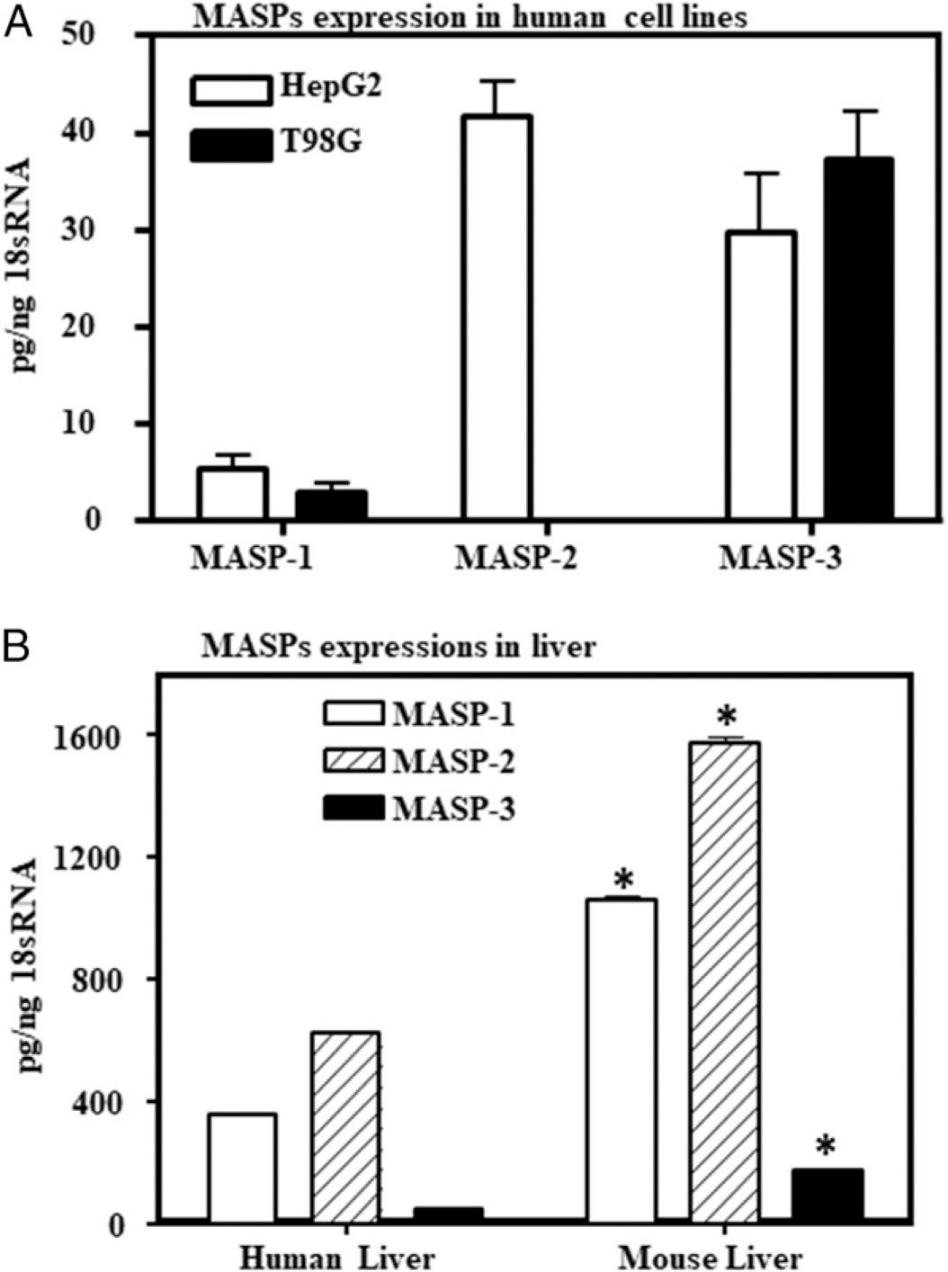
Comparing level of MASP-1, MASP-2, and MASP-3 expression by qRT-PCR in human liver and brain cell lines and in human and mouse liver. **(A)** HepG2 and T98G cell lines **(B)**. Comparing the MASP-1, MASP-2, and MASP-3 expression in the liver from mouse and human. The mRNA expression data shown in this study for MASP-1, MASP-2, and MASP-3 were repeated three times for human and mouse liver. Data are shown as mean ± SEM of three replicates. The *p* values were calculated using t test. *The *p* values <0.05 were compared between T98G and HepG2 cell lines and also between human and mouse liver as shown in the graphs.

We examined two human cell lines (i.e., T98G and HepG2 for MASP mRNA expression). Overall, human HepG2 liver cells expressed MASP-1, MASP-2, and MASP-3, and T98G cells expressed MASP-1 and MASP-3 but nodetectable MASP-2 (Fig. 1A). Human and mouse liver expressed all three mRNAs (Fig. 1B). Human liver HepG2 and brain T98G cells expressed more MASP-3 than MASP-1 mRNA (Fig. 1A). Mouse liver tissue showed significant (*p* = 0.05) higher expression levels of all MASP mRNAs (i.e., MASP-1, MASP-2, and MASP-3 expression) compared with the human liver (Fig. 1B). We used both HepG2 and T98G cells to test our ability to decrease human MASP-3 mRNA expression in a specific fashion.

### hu shMASP-3 RNA specifically decreases the expression of MASP-3 in human HepG2 and T98G cells

**FIGURE 2.**
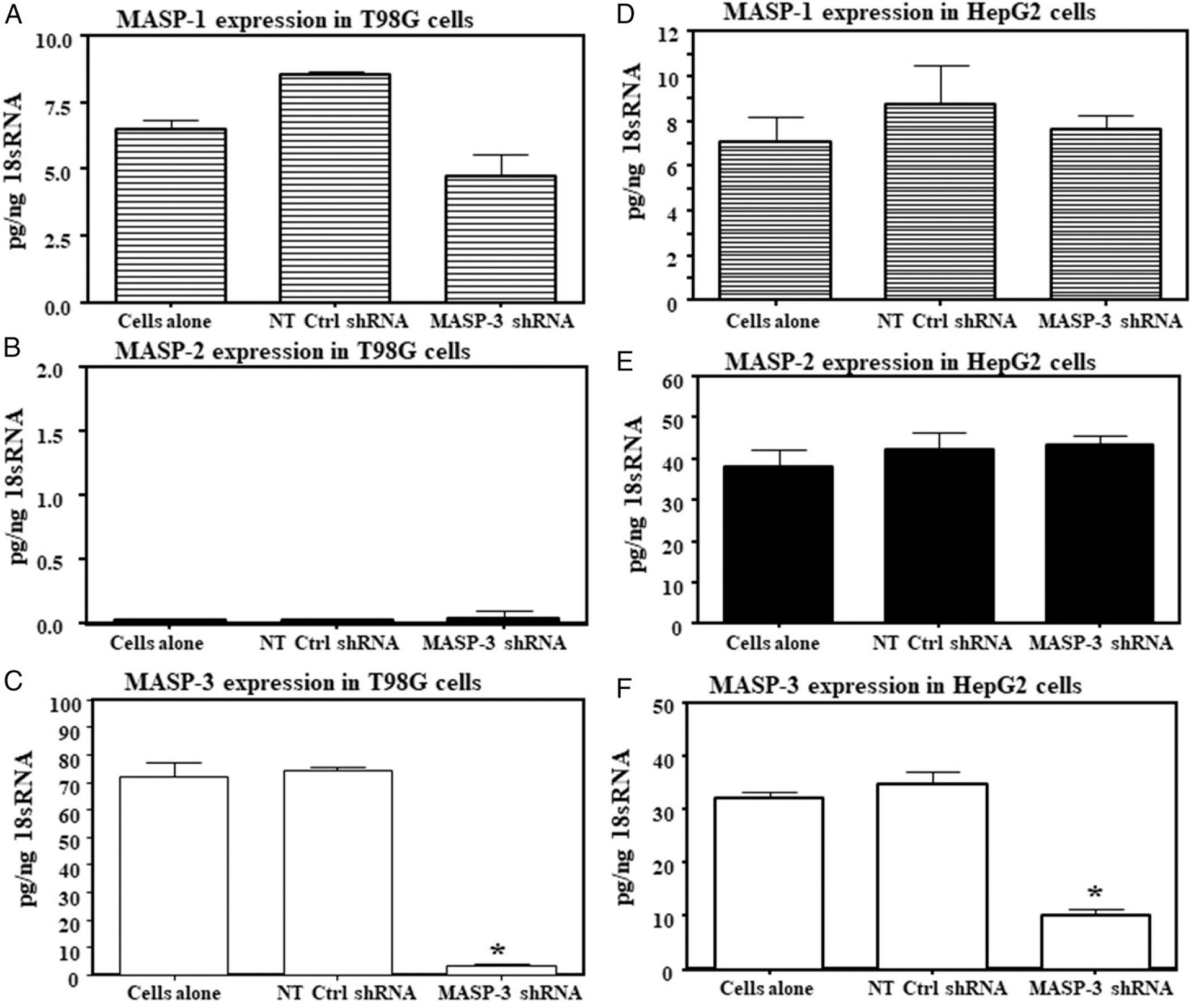
Long-term effect of the lentiviral vector–mediated RNAi by qRT-PCR on MASP-1, MASP-2, and MASP-3 expression in vitro in human T98G and HepG2 cells. T98G or HepG2 cells were either untreated (cell alone) or transfected with shRNA lentivirus carrying no-target control shScramble RNA (NT Ctrl shRNA) or with MASP-3–specific shRNA lentivirus (shMASP-3 RNA) particles. **(A)** MASP-1 expression in T98G cells. **(B)** MASP-2 expression in T98G cells. **(C)** MASP-3 expression in T98G cells. **(D)** MASP-1 expression in HepG2 cells. **(E)** MASP-2 expression in HepG2 cells. **(F)** MASP-3 expression in HepG2 cells. 18S rRNA was used an internal control. Data are shown as mean ± SEM of three replicates. The *p* values were calculated using ANOVA. *p < 0.05 versus with the cells alone or NT Ctrl shRNA or shMASP-3 RNA.

Lentiviruses expressing either GFP, hu shMASP-3 RNA, or a control hu shScramble RNA were prepared as described in Materials and Methods. Efficiency of infection was first qualitatively assessed using GFP expression. Lentiviral particles consisting of a mixture of GFP and either hu shMASP-3 RNA or control shRNA were incubated with HepG2 or T98G cells for 24 h followed by washing and feeding with fresh media. Puromycin was added to the media to select for infected cells. GFP expression was visualized aftereither24 h or 15 d in T98G cells (Supplemental Fig. 1). The percentage of T98G cells expressing GFP increased with lentiviral doses of 0.2, 0.4, and 1.6 × 10^3^ particles (data not shown). For this reason, all subsequent experiments in this series were performed with 1.6 × 10^3^ particles using HepG2 or T98G cells. After lentiviral infection, day 15 cultures from HepG2 and T98G were then examined for expression of MASP-1, MASP-2, and MASP-3 mRNA. Cultures were lysed, total RNA purified, and MASP mRNA levels measured as described in Materials and Methods. MASP-1, an alternative splice variant of the MASP-1/3 gene, was unaffected by infection with either control or shMASP-3 RNA lentiviruses (Fig. 2A, 2D). Similarly, MASP-2 gene expression was unaffected in HepG2 cells (Fig. 2E), but the direct effect of shMASP-3 RNA could not be determined on T98G cells, for these cells expressed very low or undetectable MASP-2 (Fig. 2B). In HepG2 cells, there was a significant (*p* < 0.001) 70% decrease at day 15 in the expression of MASP-3 by hu shMASP-3 RNA compared with cells alone or control Scramble shRNA (Fig. 2F). In striking contrast, in T98G cells, shMASP-3 RNA–expressing lentiviruses (but not the control lentivirus) caused the level of MASP-3mRNAtodropby 95%(Fig.2C). Nofurtherdecreaseinthe expression of MASP-3 was seen by using higher doses of lentivirus (data not shown). These data show that human MASP-3 can be silenced specifically by using hu shMASP-3 RNA in human cell lines without significantly affecting the expression of MASP-1 or MASP-2, if these are present.

### hu shMASP-3 RNA attenuated the MASP-3 protein levels in the T98G cell supernatants

To establish the durability of shMASP-3 RNA infection, T98G cells were incubated with lentiviral particles, washed, and cultured for 60 d. Supernatants were then collected, concentrated and examined for MASP-3 protein using Western blot analysis (Supplemental Fig. 2). There was an almost complete lack of MASP-3 protein in the supernatants of T98G cells at day 60 (Supplemental Fig. 2A, lane 5) compared with the cells transfected with shScramble RNA (Supplemental Fig. 2A, lane 4). Purified recombinant human MASP-3 catalytic fragment in varying amounts was used as a positive control (Supplemental Fig. 2B, lanes 2–5). These Western blot data show that hu shMASP-3 RNA specifically targeted human MASP-3 in T98G cells and reduced its expression substantially after 2 mo in culture.

### Presence of ProFD in the sera from both MASP-1/3^−/−^ mice and MASP-1/3–deficient humans

**FIGURE 3.**
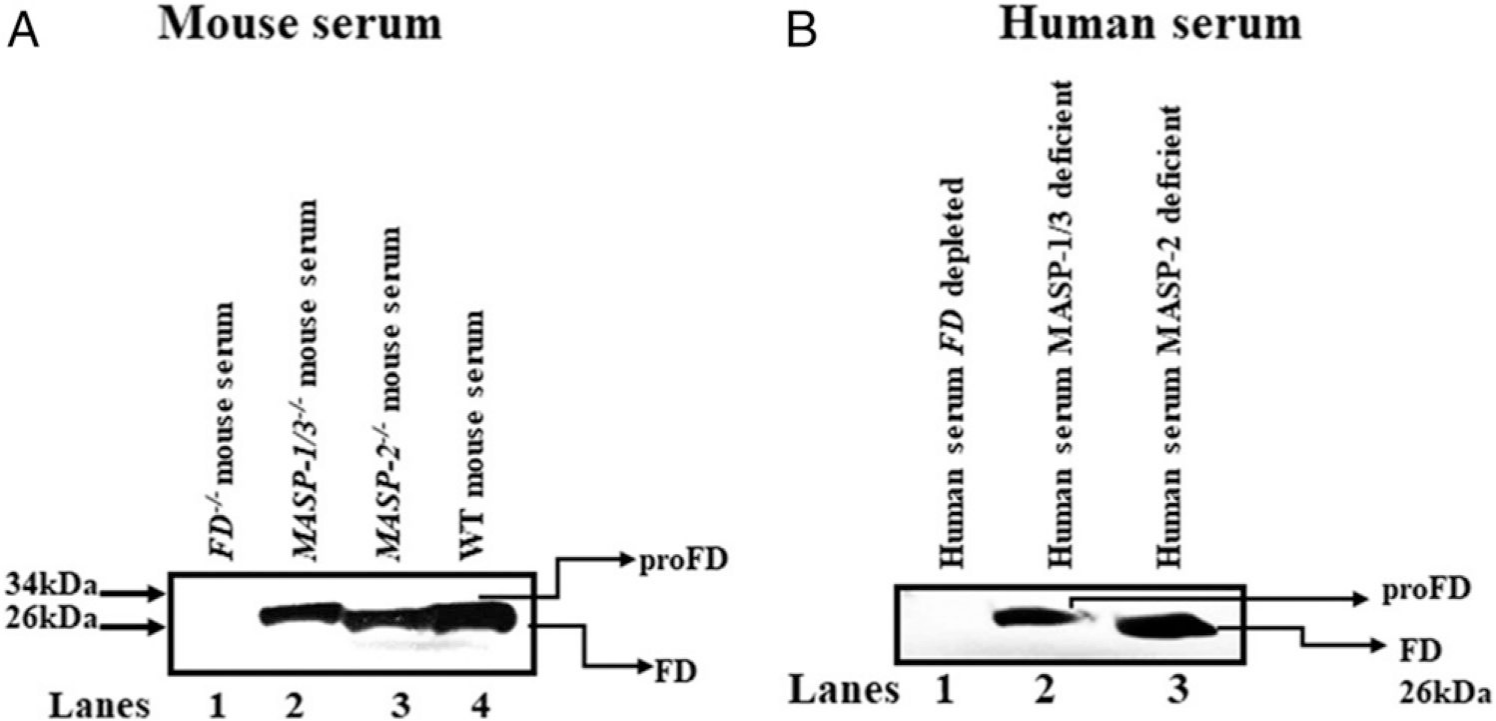
Western blot analysis for proFD or FD of human and mouse serum lacking MASP-1/3 and MASP-2 genes. **(A)** Sera from *MASP-1/3*^*−/−*^ mice contain only proFD (lane 2) versus sera from *MASP-2*^*−/−*^ mice, which contain FD (lane 3). Sera from *FD*^*−/−*^ (lane 1) and WT (lane 4) mice were used as a negative and positive controls, respectively. **(B)** Sera from human deficient in MASP-1/3 contain only proFD (lane 2) versus sera from human lacking *MASP-2*^*−/−*^, which contains FD (lane 3). Human sera depleted of FD (lane 1) were used as a negative control. These Western blot experiments were repeated three times.

To explore the role of MASP-3 in the cleavage of proDF to mature FD, we examined sera from both mice and humans deficient in the alternatively spliced MASP-1/3 gene by immunoprecipitation followed by Western blot analysis (Fig. 3). We found that both mouse and human serum contained only ProFD in the complete absence of MASP-1/3 (Fig. 3A, lane 2, and Fig. 3B, lane 2). In contrast, no proFD but only FD was present in human sera with MASP-2 deficiency (Fig. 3, lane 3). Serum from *MASP-2*^*−/−*^-deficient mice also contained FD, but did not contain proFD (Fig. 3, lane 2). Sera from *FD*^*−/−*^ were used as a negative control, and no band of either FD or proFD is present (Fig. 3A, lane 1, and Fig. 3B, lane 1).

### In vivo recombinant MASP-3 activated AP and cleaved proFD in MASP-1/3^−/−^ mice

**FIGURE 4.**
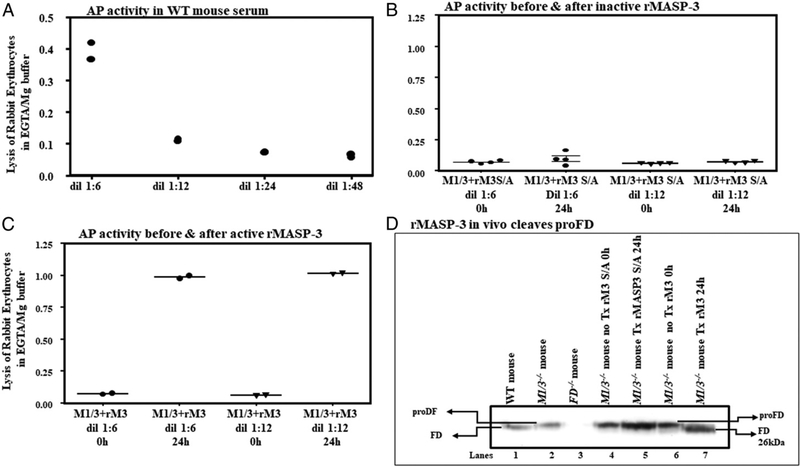
In vivo reconstitution of AP activity in *MASP-1/3*^*−/−*^ mice with rMASP-3. An ex vivo assessment of the AP by lysis of rabbit blood erythrocytes (RBEs) and cleavage of proDF by Western blot analysis at 24 h from *MASP-1/3*^*−/−*^–injected mice with active (rM3) or inactive or mutated rM3 (rM3 S/A). **(A)** Natural RBEs lysis by WT serum using various dilutions as expected. **(B)** No RBEs lysis by *MASP-1/3*^*−/−*^ mouse serum reconstituted with rM3 S/A. **(C)** RBEs lysis by *MASP-1/3*^*−/−*^ serum reconstituted with rM3. **(D)** Western blot showing the cleavage of proFD into FD only after injecting rM3, not rM3 S/A (lane 7 versus lane 5). Data from *MASP-1/3*^*−/−*^ mice shown (B and C) as mean OD ± SEM. All samples were run in duplicate with highly reproducible data from two *MASP-1/3*^*−/−*^ male and two female mice. Western blot were repeated three times. M1/3 + rM3, *MASP-1/3*^*−/−*^ mice treated active rMASP-3; M1/3 + rM3 S/A, *MASP-1/3*^*−/−*^ mice treated inactive rMASP-3; dil, serum dilutions; Tx, treatment.

To determine the requirement of MASP-3 in vivo to reconstitute the AP, *MASP-1/3*^*−/−*^ mice were injected with an active recombinant MASP-3 (rM3) or with an inactive or mutated rM3 (rM3 S/A), and AP activity was examined after 24 h by well-known rabbit erythrocyte lysis assay specific for measuring the AP activity (32). AP is normally compromised in *MASP-1/3*^*−/−*^ mice versus WT mice because of proFD presence in their circulation (Fig. 4A, 4B). Exogenous active rMASP-3 compared with the mutated rMASP-3 clearly reconstituted the AP activity in the sera from *MASP-1/3*^*−/−*^ mice at 24 h (Fig. 4C). The ex vivo AP activity in sera from *MASP-1/3*^*−/−*^ mice after reconstitution was equivalent to the WT mouse serum (Fig. 4A, 4C). There were no differences in the ability of active rM3 to reconstitute the AP activity both in male and female mice (Fig. 4C). Furthermore, at 24 h, exogenous rM3 not only activated the AP but also cleaved the proDF in vivo in *MASP-1/3*^*−/−*^ mice (Fig. 4D, lane 7 versus lane 5). These data show that MASP-3 is specifically required for the cleavage of proDF into DF in vivo and results in the subsequent capacity for AP activation.

### In vitro identification of GalNAc–MASP-3–siRNA duplexes

**FIGURE 5.**
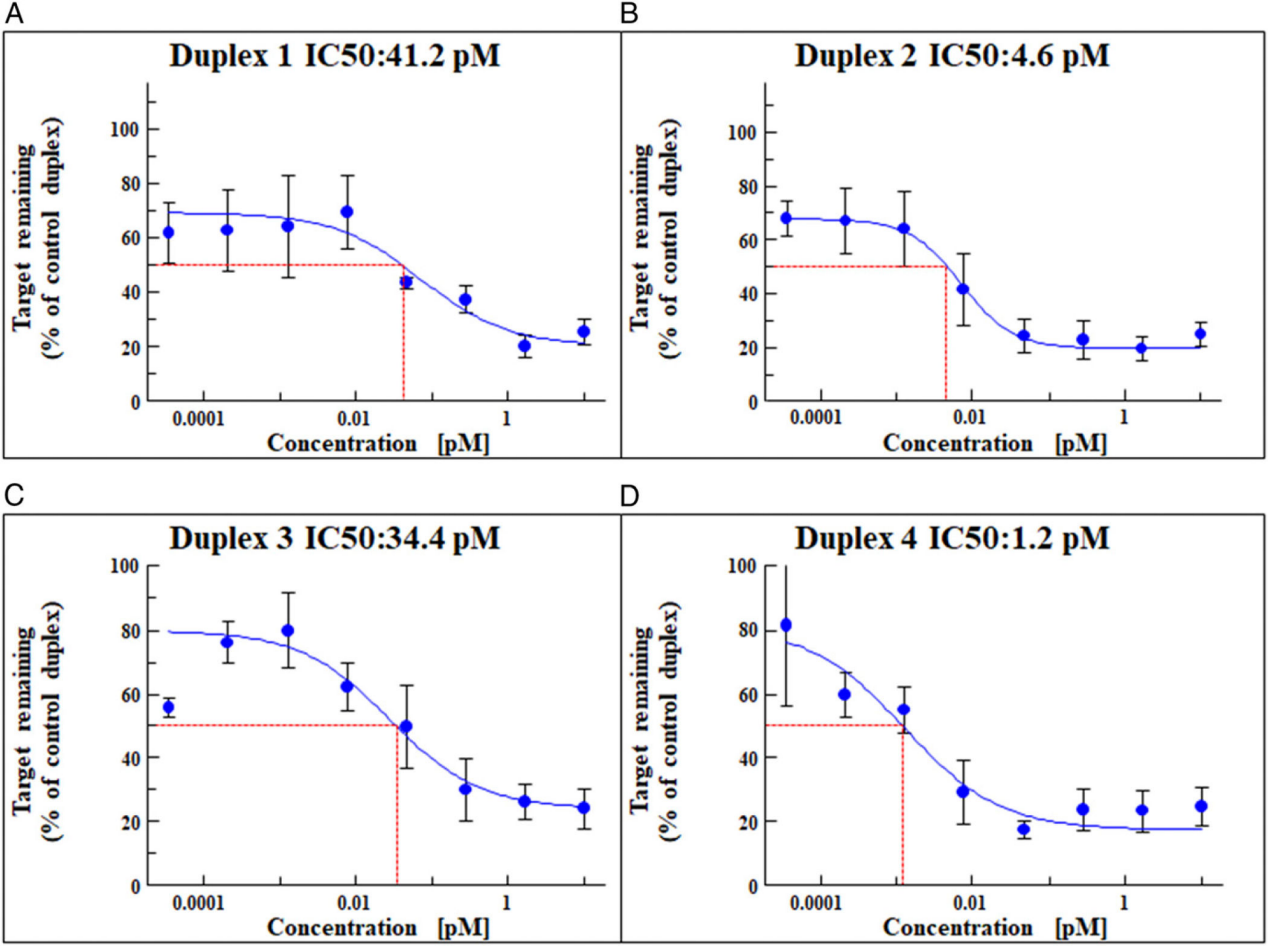
IC_50_s for MASP3 silencing with GalNAc-MASP3 siRNAs: Dual-Glo luciferase assay was performed using Cos7 cells expressing a firefly luciferase–MASP3 fusion construct. Cells were transfected with mouse MASP3-specific siRNAs at the concentrations indicated. Luminescence levels were measured at 48 h post-transfection. The ratio of normalized luminescence was plotted in Microsoft Excel to calculate the IC_50_ for each duplex. **(A)** Duplex 1 IC_50_ = 41.2 pM. **(B)** Duplex 2 IC_50_ = 4.6 pM. **(C)** Duplex 3 IC_50_ = 34.4 pM. **(D)** Duplex 4 IC_50_ = 1.2 pM. Duplex 1 = duplex 3 and duplex 2 = duplex 4. These experiments were repeated two times with each new duplex with highly reproducible results, and data are shown as mean ± SEM. IC_50_, half maximum inhibitory concentration.

Mouse-specific siRNA sequences were identified using Alnylam bioinformatic tools. Chemically stabilized duplexes conjugated to a triantennary GalNAc on the 3’ end of the sense strand were synthesized using solid-phase synthesis methods (46). MASP-3–siRNAs were tested in vitro using a psiCHECK vector expressing MASP3 gene in Cos-7 cells. The silencing of MASP-3 was examined based on the inhibition of luciferase activity following transduction with various GalNAc–MASP-3–siRNA duplexes. Four duplexes show IC_50_ silencing of 41.2, 4.6, 34.4, and 36 pM, respectively (Fig. 5).

### GalNAc–MASP-3–siRNA duplex silenced MASP-3 expression in mice with no disease

**FIGURE 6.**
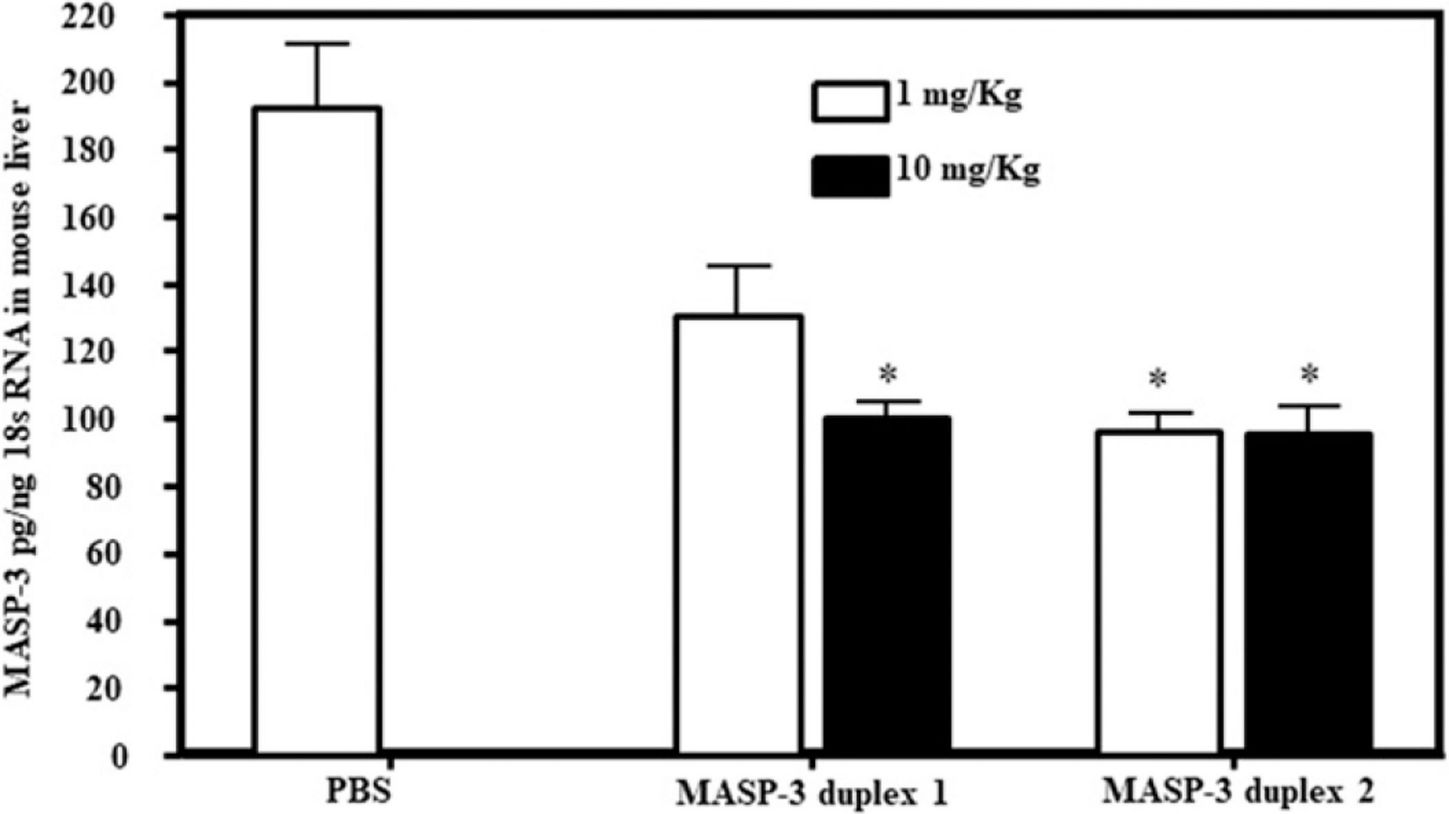
Semiquantitative RT-PCR analysis of MASP-3 expression from the liver of WT mice injected with GalNAc–MASP-3–siRNA duplexes. Total RNA was extracted at day 7 from the liver of mice injected s.c. with two different doses of GalNAc–MASP-3–siRNA duplexes. MASP-3 expression was examined using qRT-PCR. Liver from WT mouse was used as a positive control to make a standard curve (data not shown). 18s RNA was used as an internal control to calculate the expression of MASP-3 (pg/18s RNA). Data shown as mean ± SEM of three replicates. Empty bars = 1 mg/kg and solid black bars = 10 mg/kg. The *p* values were calculated using t test. *p < 0.05 versus PBS.

Three independent in vivo experiments were done to determine the efficiency of GalNAc–luciferase–siRNA or GalNAc–MASP-3–siRNA duplexes on the expression of MASP-3. In the first experiment, to examine the short-term effect of GalNAc–MASP-3, WT mice (*n* = 15) were injected s.c. with a single dose of either PBS or GalNAc–MASP-3 duplexes 1 or 2 at day 0 and sacrificed at day 7 (Fig. 6). Duplex 2 was most effective at blocking MASP-3 at both 1 and 10 mg/kg doses. Both duplexes have no effect on the expression of MAp44 (Supplemental Fig. 3A) and MASP-1 (Supplemental Fig. 3B).

**FIGURE 7.**
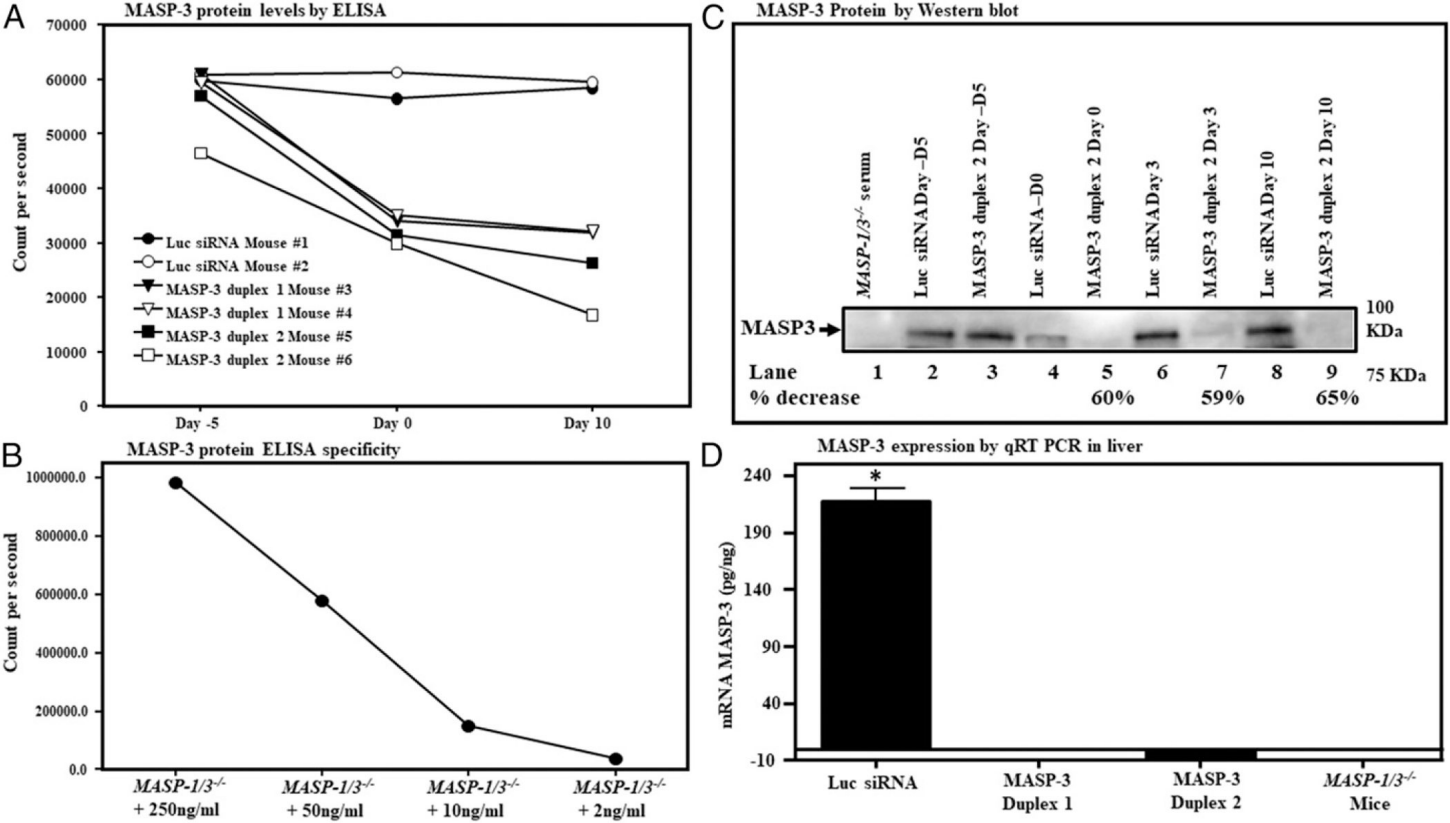
A short-term comparison of the in vivo efficiency of GalNAc–MASP-3–siRNA duplex 1 or GalNAc–MASP-3–siRNA duplex 2 in mice with no CAIA. **(A)** ELISA showing the levels of MASP-3 protein in the sera from six mice treated with GalNAc–luciferase–siRNA (*n* = 2) (mouse no. 1 and mouse no. 2) or GalNAc–MASP-3–siRNA no. 1 (mouse no. 3 and mouse no. 4) or GalNAc–MASP-3–siRNA no. 2 duplexes (mouse no. 5 and mouse no. 6). **(B)** A standard curve showing the specificity of MASP-3 protein ELISA using serum from a MASP-1/3–deficient mouse reconstituted with various doses of active recombinant mouse MASP-3 (250, 50, 10, and 2 ng/ml). **(C)** Western blot analysis for MASP-3 protein levels using sera from WT mice (mouse no. 1 or mouse no. 6) injected with GalNAc–luciferase–siRNA or with GalNAc–MASP-3–siRNA no. 2 duplex, at day −5 (lanes 2 and 3), at day 0 (lanes 4 and 5), at day 3 (lanes 6 and 7), and at day 10 (lanes 8 and 9). Sera from *MASP-1/3*^*−/−*^ mice with no injections of siRNAs were used as a negative control (lane 1). Western blot data were repeated two times from mouse no. 1 and mouse no. 6. A band of MASP-3 protein (~94–100 kDa) in serum shows its presence. **(D)** qRT-PCR showing the expression of MASP-3 in the liver at day 10 from WT mice injected with MASP-3 no. 1 or no. 2 duplexes. Western blots were replicated two times. Mean ± SEM of three replicates. Error bars in the second and third bars are invisible because of very low values in mice treated with GalNAc–MASP-3–siRNA duplexes no. 1 or 2. The *p* values were calculated using t test. *p < 0.05 comparing Luc siRNA–treated mice with GalNAc–MASP-3–siRNA duplex 1–treated or duplex 2–treated mice. Luc, luciferase.

In the second experiment, to examine the long-term effect of GalNAc–MASP-3–siRNA, WT mice (*n* = 6) were injected s.c. three times either with GalNAc–luciferase–siRNA or GalNAc–MASP-3–siRNA duplexes 1 or 2 at day −5, at day 0, and at day 3. The mice were then sacrificed at day 10 (Fig. 7). ELISA results confirmed that at day 10, there was a decrease in the levels of MASP-3 protein, which ranged from 54 to 64% of control in mice injected with GalNAc–MASP-3–siRNA duplex 2 (Fig. 7A). A standard curve was generated after reconstituting the sera from *MASP-1/3*^*−/−*^ mice with known quantities of rM3 to show the specificity of MASP-3 ELISA (Fig. 7B). Sera from these mice were also analyzed by Western blot, confirming decreases in the range of 60% as measured by densitometric analysis (Fig. 7C, lane 7). Additionally, duplexes 1 and 2 caused a marked decrease in liver MASP-3 mRNA abundance by day 10 as compared with control (Fig. 7D).

**FIGURE 8.**
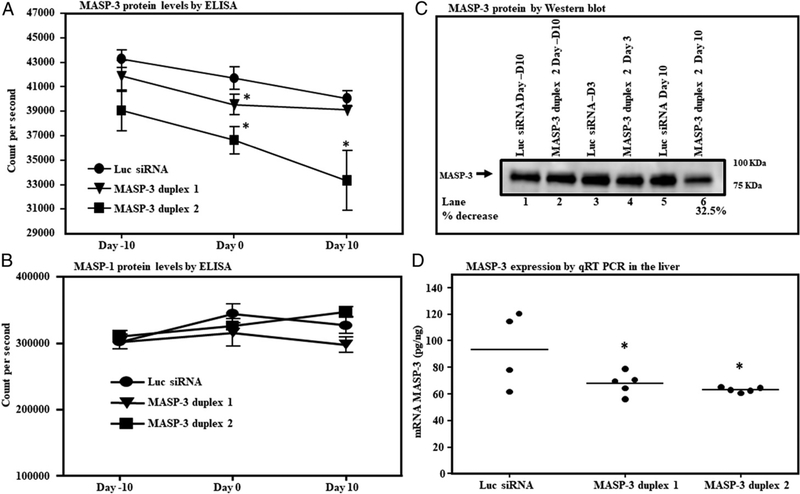
A long-term comparison of the in vivo efficiency of GalNAc–MASP-3–siRNA duplex 1 or GalNAc–MASP-3–siRNA duplex 2 in mice with no CAIA. **(A)** ELISA showing a decrease in the levels of MASP-3 protein in the sera from mice treated with GalNAc–MASP-3–siRNA no. 1 or no. 2 duplex. **(B)** ELISA showing no off-target effect on the levels of MASP-1 protein from mice injected with GalNAc–MASP-3–siRNA duplex 1 and GalNAc–MASP-3–siRNA duplex no. 2 or GalNAc–luciferase–siRNA. **(C)** Western blot analysis for MASP-3 protein using sera from WT mice injected only with GalNAc–MASP-3–siRNA duplex no. 2. MASP-3 protein in the sera from WT mice injected with GalNAc–uciferase–siRNA or GalNAc–MASP-3–siRNA duplex no. 2, at day −10 (lanes 1 and 2), day 3 (lanes 3 and 4), and at day 10 (lanes 5 and 6). **(D)** qRT-PCR showing the expression of MASP-3 in the liver at day 10 from mice injected with GalNAc–luciferase–siRNA or GalNAc–MASP-3–siRNA duplexes no. 1 or 2. 18s RNA was used as an internal control and to calculate the expression of MASP-3 in the liver. Data from all mice have been included. The *p* values were calculated using *t* test. *p < 0.05 versus mice injected with Luc siRNAs were compared using *t* test. Luc, luciferase.

In the third experiment, WT mice (*n* = 15) were injected three times with GalNAc–luciferase–siRNA or GalNAc–MASP-3–siRNAs duplex 1 or duplex 2 at day −10, day 0, and at day 3 followed by sacrificing all mice at day 10 (Fig. 8). In this fashion, the time between first and second doses was extended from 5 to 10 d. Serum MASP-3 dropped by ~15% by day 10 (*p* = 0.015) as compared to the 60% drop seen in the previous experiment (compare Figs. 7A and 8A). MASP-1 protein levels were completely unaffected (Fig. 8B). Sera from mice injected with duplex 2 were also analyzed by Western blot (Fig. 8C). Densitometry measured the decrease in MASP-3 to be 32.5% at day 10. Liver from mice injected with duplex 1 or duplex 2 of GalNAc–MASP-3–siRNA also showed a 33.0% (*p* = 0.044) decrease in the expression of MASP-3 after treatment (Fig. 8D). These short-term and long-term experiments in healthy WT mice show that duplex 2 of GalNAc–MASP-3–siRNA performed better compared with duplex 1; therefore, we decided to use duplex 2 for CAIA study.

### GalNAc–MASP-3–siRNA duplex partially attenuated clinical disease in mice with CAIA

**FIGURE 9.**
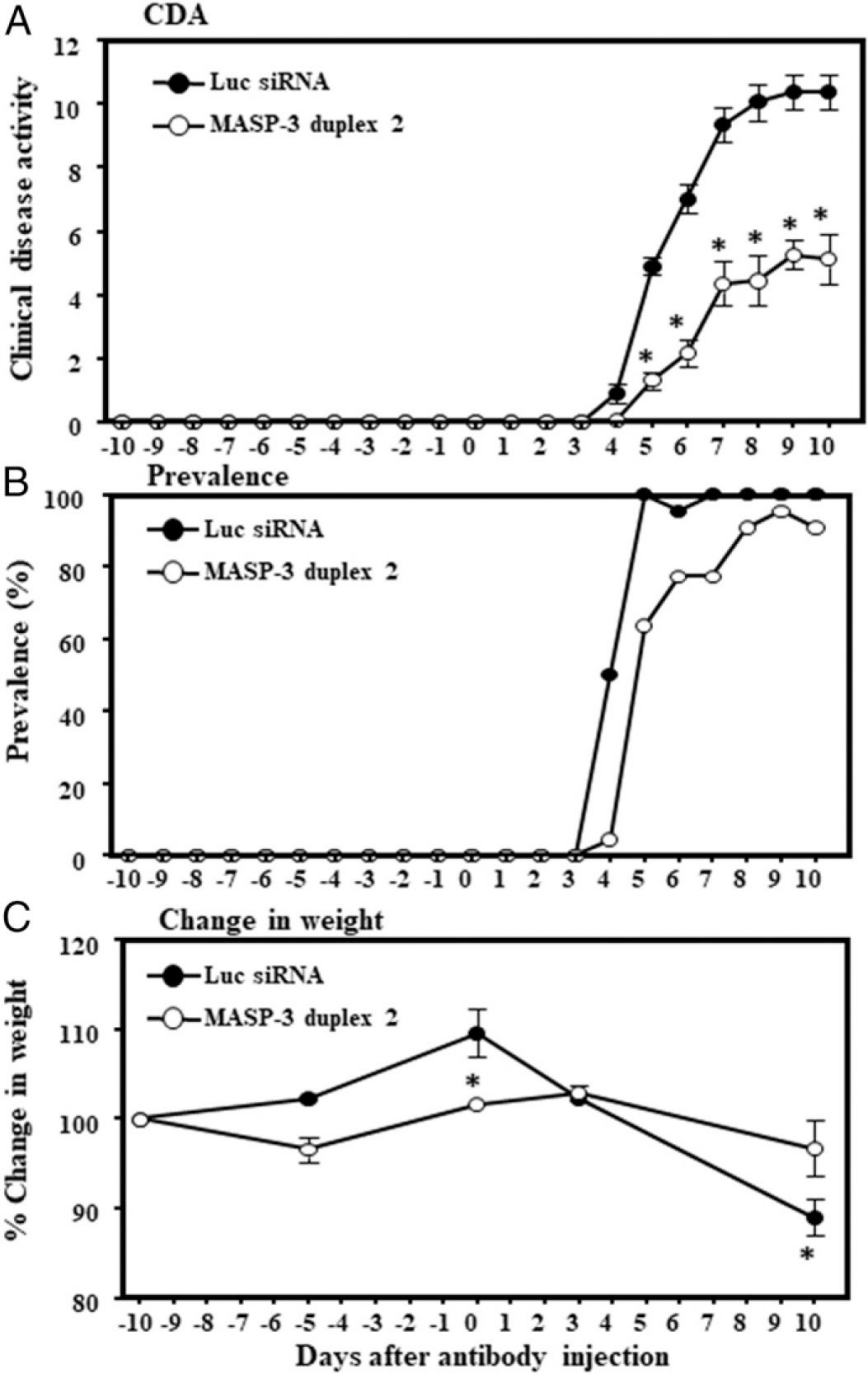
CDA in WT mice treated with GalNAc–MASP-3–siRNA duplex 2. **(A)** CDA in mice treated with GalNAc–luciferase–siRNA or GalNAc–MASP-3–siRNA. **(B)** Prevalence (%) of disease at day 10 in mice treated with GalNAc–luciferase–siRNA or GalNAc–MASP-3–siRNA duplex 2. **(C)** Change in weight (%) in mice treated with GalNAc–luciferase–siRNA or GalNAc–MASP-3–siRNA duplex 2. Data shown represent the mean ± SEM based on WT mice injected s.c. with GalNAc–luciferase–siRNA, *n* = 22 and with GalNAc–MASP-3–siRNA duplex 2, *n* = 22. In vivo CAIA experiments were repeated three separate times using total *n* = 44 (cohort 1 *n* = 14 mice, cohort 2 *n* = 14 mice, and cohort 3 *n* = 16 mice). The *p* values were calculated using t test. *p < 0.05 in comparison with mice to GalNAc–luciferase–siRNA–injected mice.

To examine the efficacy of our GalNAc–MASP-3–siRNA duplex 2 for the treatment of RA, we employed the CAIA model in which mice are injected with pathogenic anti-collagen Abs on day 0 and then injected with LPS on day 3. Synovitis develops rapidly and peaks by day 10. Mice are then sacrificed on day 10 for analysis. In this experiment, WT mice (*n* = 22) were injected s.c. on day −10 beforetheinductionofdiseaseandagainonday–5andonday0with either GalNAc–luciferase–siRNA or with GalNAc–MASP-3–siRNA (duplex 2) (*n* = 22) as described in Materials and Methods (Fig. 9). CDA was assessed daily. Strikingly, initial disease progression in the MASP-3 siRNA group was markedly slowed as compared with the luciferase siRNA–injected group until day 6 where the CDA was decreased by 69% (*p* < 0.001). Between days 6 and 7, there was a reproducible acceleration in disease in the MASP-3 siRNA injection group followed by a general disease plateau for both groups. By day 10, the average CDA for the luciferase siRNA–injected group was 10.40 ± 0.545, whereas the average CDA for the MASP-3 siRNA–injected group was 5.13 ± 0.798; thus, a decrease of 50% was observed (*p* < 0.001) (Fig. 9A). In all three separate experiments, there was a consistent decrease in the CDA in mice treated with MASP-3 siRNA. Individual CDA in each mouse was also consistent with the overall decrease in the CDA in mice treated with GalNAc–MASP-3–siRNA (data not shown). The prevalence of disease at day 10 in WT mice injected with GalNAc–luciferase–siRNA or GalNAc–MASP-3–siRNA duplex 2 was 100 and 90%, respectively (Fig. 9B). There was a significant (*p* = 0.05) effect on the weight of mice treated with GalNAc–luciferase–siRNA or GalNAc–MASP-3–siRNA duplex 2 at day 0 and at day 10 (Fig. 9C). All mice survived to the experimental endpoint, and these in vivo CAIA were repeated three times (*n* = 44) with almost similar results; data shown in this paper are from all experiments. Overall, these data indicate that GalNAc–MASP-3–siRNA duplex 2 partially inhibited the development of CAIA in mice.

### Decrease in the histopathology scores, C3 deposition, and macrophages in the joints of mice treated with GalNAc–MASP-3–siRNA

**FIGURE 10.**
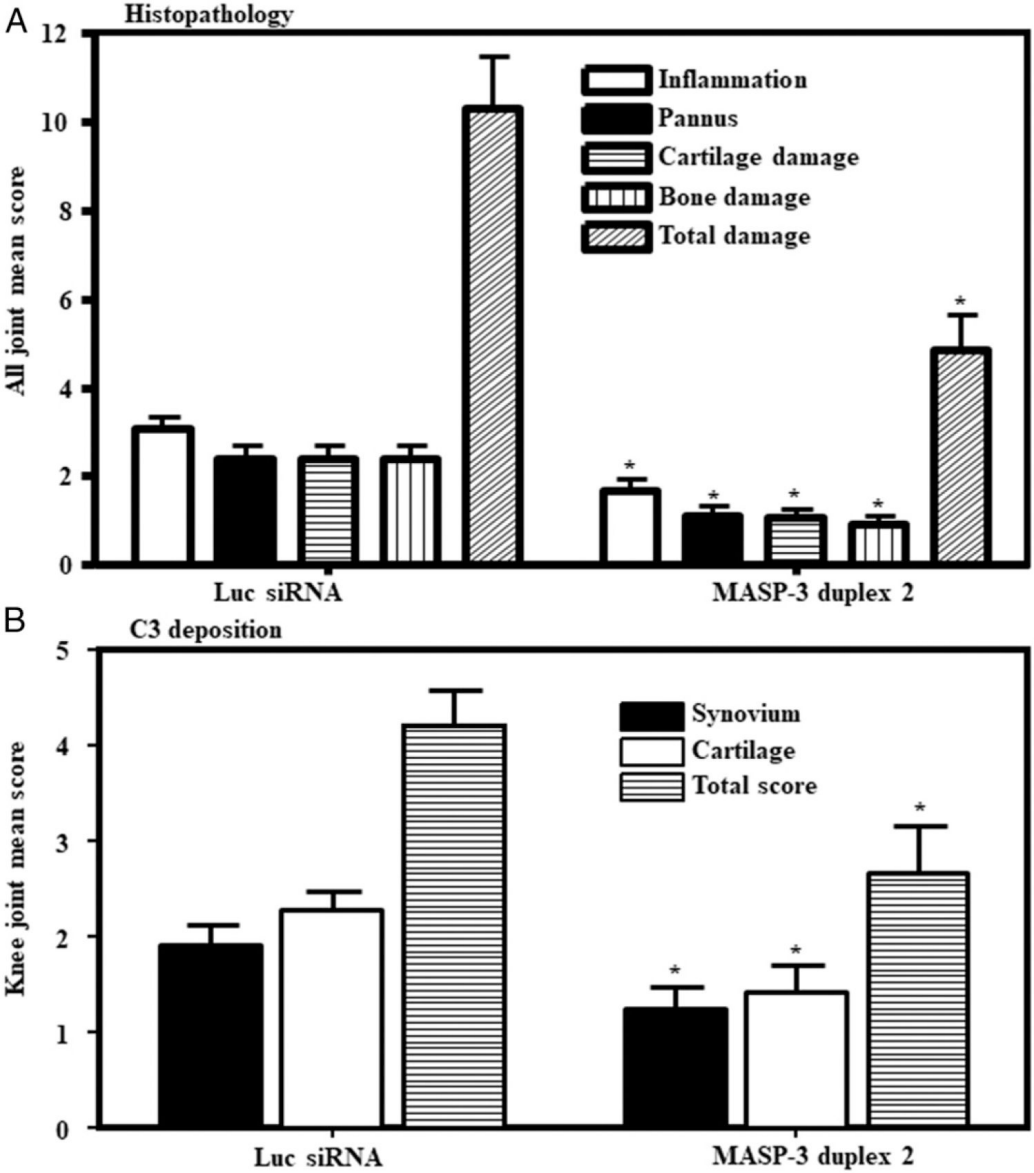
Histopathology scores and C3 deposition in CAIA mice injected s.c. with GalNAc–MASP-3–siRNA duplex 2 compared with GalNAc–luciferase–siRNA. All mice were sacrificed at day 10 and histopathology using T blue measured in AJM score at day 10. **(A)** AJM histopathology for inflammation, pannus formation, cartilage damage, and bone damage. **(B)** Mean C3 deposition scores from both the knee joints in the synovium, on the surface of cartilage, and total scores (synovium plus cartilage). All data represent the mean ± SEM comparing GalNAc–luciferase–siRNA–treated mice with GalNAc–MASP-3–siRNA duplex 2. The *p* values were calculated using ANOVA. *p < 0.05 in comparison with the mice injected with GalNAc–luciferase–siRNA.

**FIGURE 11.**
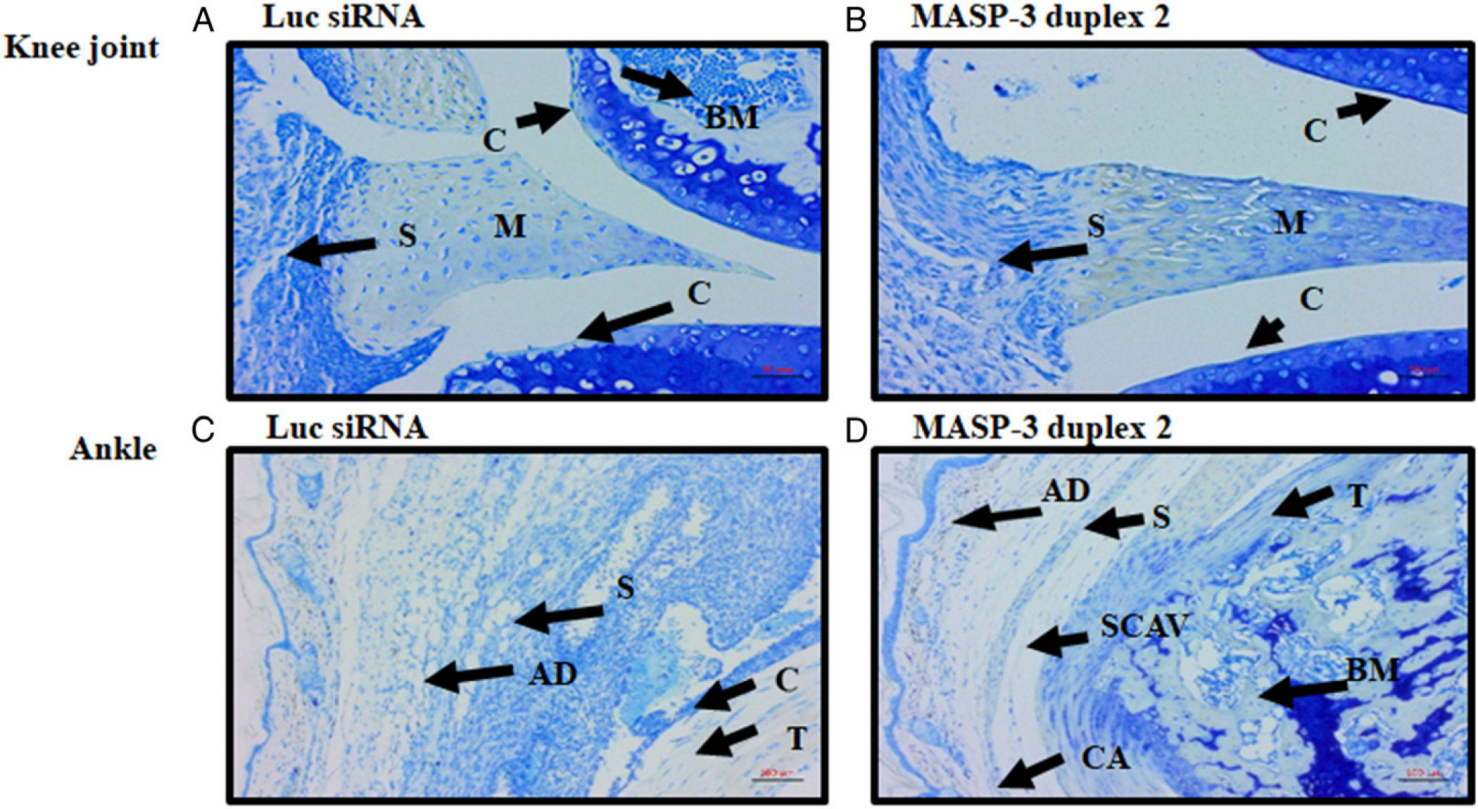
Representative histopathology images from the knee joints and ankle of CAIA mice injected s.c. with GalNAc–MASP-3–siRNA duplex 2 or GalNAc–luciferase–siRNA. The top two panels from left to right (**A** and **B**) show staining with T blue (blue color) from the knee joints of CAIA mice treated with GalNAc–luciferase–siRNA (left panel) or with GalNAc–MASP-3–siRNA duplex 2 (right panel). The second set of two panels from left to right (**C** and **D**) show staining with T blue from the ankle joints of CAIA mice treated with GalNAc–luciferase–siRNA (left panel) or with GalNAc–MASP-3–siRNA duplex 2 (right panel). Areas of synovium (S, arrow), cartilage (C, arrow), bone marrow (BM, arrow), adipose tissue (AD, arrow), synovial cavity (SCAV, arrow), calcaneus (CA, arrow), talus (T, arrow), and meniscus (M, arrow) are identified. The sections were photographed at original magnification ×20. Scale bars shown in red in the bottom right of each panel for knee joint (A and B), 0.05 mm (50 μm, original magnification ×20), and for ankle (C and D), 0.1 mm (100 μm, original magnification ×10).

Joints derived from the CAIA experiments no. 1 (*n* = 14) out of three experiments described above were collected for further analysis. All five joints (i.e., both forelimbs and the right hind limb) (knee joint, ankle and hind paw) were processed for histopathology studies and for the measurement of C3 deposition in the joints (Fig. 10A). All data are shown as an all joint mean (AJM) score (0–5). Histopathology scores for inflammation, pannus formation, cartilage damage, bone damage show a 53% significant (*p* = 0.006) decrease (Fig. 10A). These data are consistent with the improvement in CDA (Fig. 9A). There was a significant decrease in inflammation 45% (*p* = 0.007), pannus formation 53% (*p* = 0.006), cartilage damage 55% (*p* = 0.0049) and bone damage 61% (*p* = 0.0019) in CAIA mice treated with GalNAc–MASP-3–siRNA duplex 2 compared with CAIA mice treated with GalNAc–luciferase–siRNA (Fig. 10A). Representative pictures shown of T blue staining related to histopathology from the knee joints (Fig. 11A, 11B) and ankle (Fig. 11C, 11D) of mice treated with GalNAc–MASP-3–siRNA duplex 2 or GalNAc–luciferase–siRNA.

**FIGURE 12.**
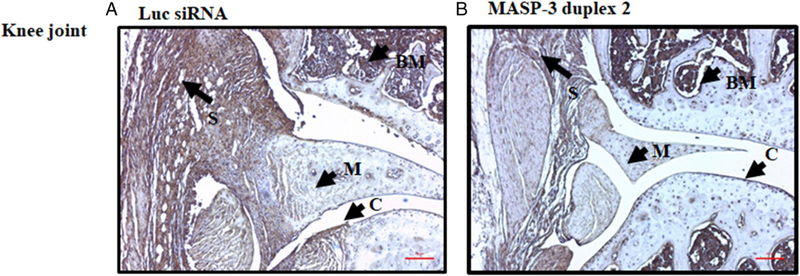
Representative C3 deposition images from the knee joints of CAIA mice injected s.c. with GalNAc–MASP-3–siRNA duplex 2 or GalNAc–luciferase–siRNA. The two panels from left to right (**A** and **B**) show IHC C3 staining using anti-C3 Ab (brown color) from the knee joints of CAIA mice treated with GalNAc–luciferase–siRNA (left panel) or with GalNAc–MASP-3–siRNA duplex 2 (right panel). Areas of synovium (S, arrow), cartilage (C, arrow), bone marrow (BM, arrow), and meniscus (M, arrow) are identified. The sections were photographed at original magnification ×20. Scale bar shown in red in the bottom right in (A) and (B) for knee joint, 0.1 mm (100 μm).

Both the knee joints were examined for C3 deposition in the synovium as well as on the surface of cartilage (Fig. 10B). All data are shown as an AJM score (0–4) and also the total score (0–8). There was a 35% (*p* = 0.031) and 37% (*p* = 0.017) decrease in the C3 deposition in the synovium and also on the surface of cartilage, respectively (Fig. 10B), in mice treated with GalNAc–MASP-3–siRNA duplex 2 (*n* = 7) compared with mice treated with GalNAc-luciferase. These data are also consistent with the decrease in CDA in mice treated with GalNAc–MASP-3 versus GalNAc-luciferase (*n* = 7). Representative pictures shown of C3 deposition from the knee joints (Fig. 12A, 12B) of mice treated with GalNAc–MASP-3–siRNA duplex 2 or GalNAc–luciferase–siRNA.

**FIGURE 13.**
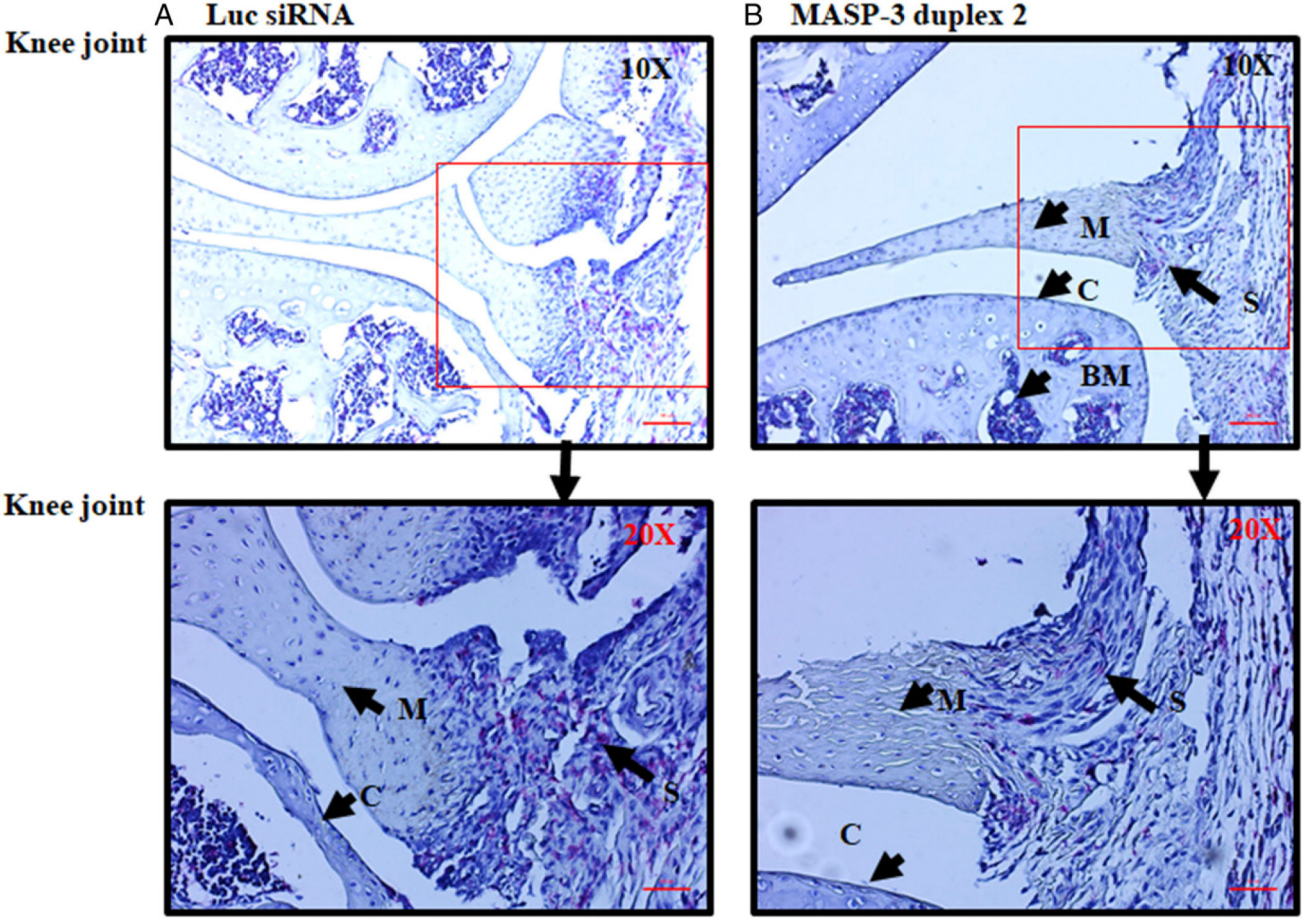
Representative macrophage staining images only from the knee joints of CAIA mice injected s.c. with GalNAc–MASP-3–siRNA duplex 2 or GalNAc–luciferase–siRNA. The two panels from left to right (**A** and **B**) show IHC macrophage staining using anti-F4/80 Ab (red color) from the knee joints of CAIA mice treated with GalNAc–luciferase–siRNA (left panel) or with GalNAc–MASP-3–siRNA duplex 2 (right panel). The two panels are showing the enhanced (original magnification ×20) red boxed area of the synovium for clarity. Areas of synovium (S, arrow), cartilage (C, arrow), bone marrow (BM, arrow), and meniscus (M, arrow) are identified. The sections were photographed at original magnification ×10. Scale bars shown in red in the bottom right of each panel for knee joint, original magnification ×20, 0.05 mm (50 μm), and original magnification ×10, 0.1 mm (100 μm).

There was a nonsignificant 28% decrease (*p* = 0.39) in macrophage infiltration in the synovium of knee joints of mice with CAIA treated with GalNAc–MASP-3–siRNA duplex 2 compared with mice treated with GalNAc-luciferase. Representative pictures shown of macrophage staining from the knee joints (Fig. 13A, 13B) of mice with CAIA treated with GalNAc–MASP-3–siRNA duplex 2 or GalNAc–luciferase–siRNA.

### Decrease in MASP-3 protein deposition in the joints of mice after treatments with GalNAc–MASP-3–siRNA

**FIGURE 14.**
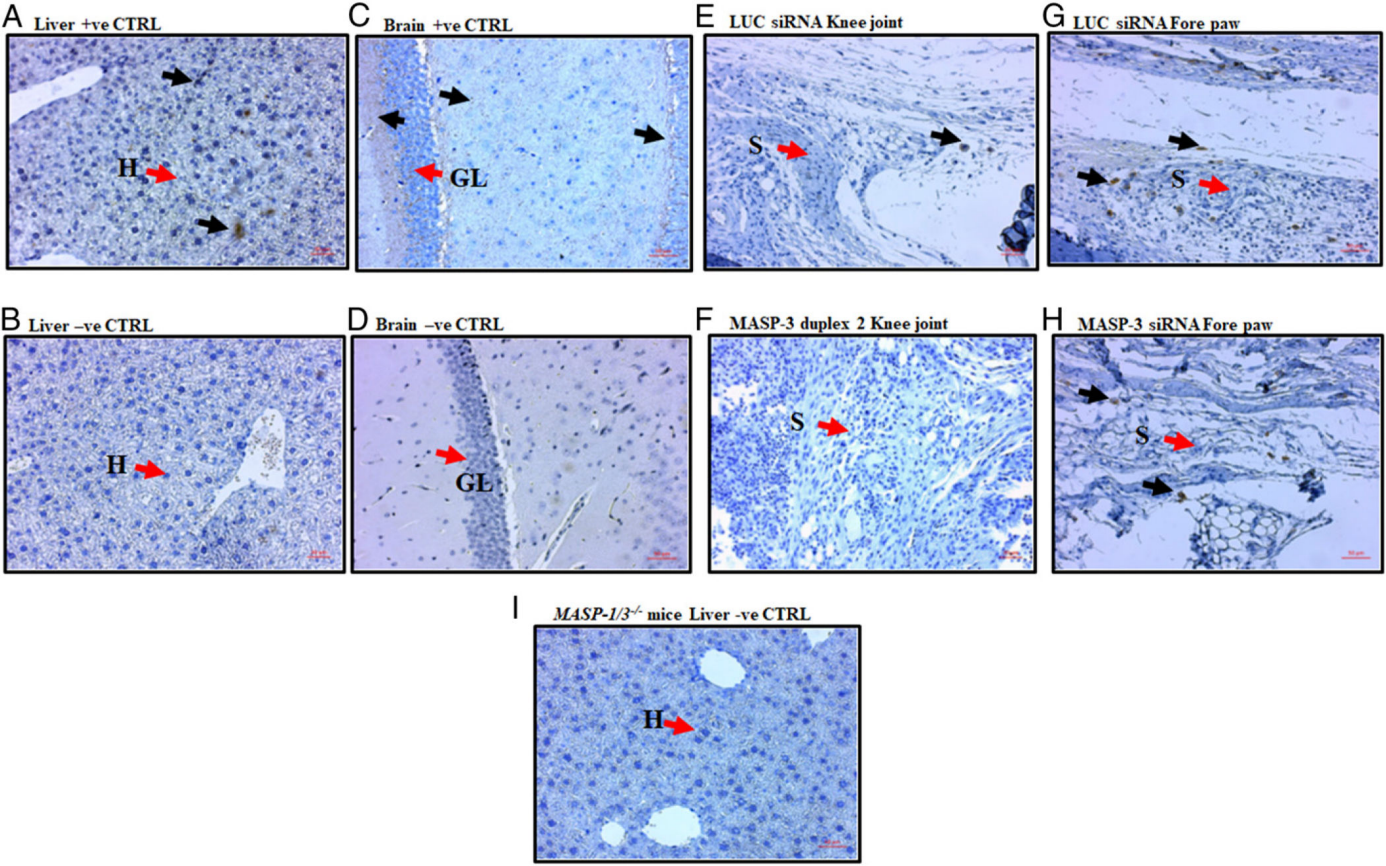
IHC localization of MASP-3 protein in joints in mice with arthritis. Representative images of the joints from WT with CAIA-treated GalNAc–Luc–siRNA and GalNAc–MASP-3 siRNA. The top set of four panels from left to right (**A**, **C**, **E**, and **G**) show positive staining with biotinylated anti-mouse MASP-3 (dark brown color) (black arrow) from the liver (first panel), brain (second panel), knee joints (KJ) (third panel), and forepaws (FP) (last leftmost panel) of WT mice. The hepatocytes (H) (red arrow) present in the liver were positive for MASP-3, whereas brain (i.e., granular layer cells [GL]) were all strongly positive for MASP-3 (A and C). Many cells were positive for MASP-3 protein in the synovium (S) from KJ and FP in WT mice with CAIA treated with GalNAc–Luc–siRNA. The middle set of four panels from left to right (**B**, **D**, **F**, and **H**) show no staining (no dark brown color) used as negative controls by staining using diluent alone without primary Ab from the liver (first middle panel) and brain (second middle panel) of WT mice. There were no or few MASP-3–positive cells in the KJ (third middle panel) and FP (last middle rightmost panel) of WT mice with CAIA treated with GalNAc–MASP-3–siRNA. The single bottom panel shows no MASP-3 staining (no dark brown color) (negative control) from the liver of *MASP-1/3*^*−/−*^ mice (**I**) (bottom panel) as expected. The liver, brain, KJ, and FP sections stained for MASP-3 deposition were counterstained with H&E and photographed at original magnifications ×20 or ×40 using Zeiss Observer. D1 (Axio) microscope. Joint sections were examined in duplicate from WT (*n* = 7) with CAIA treated with GalNAc–MASP-3–siRNA and from WT with CAIA treated with GalNAc–luciferase–siRNA, but representative pictures from each treatment groups have been shown. Red scale bar shown in (A), (B), (E), and (F), 20 μm. Red scale bar in (C), (D), (G), (H), and (I), 50 μm. CTRL, control. LUC, luciferase.

IHC analysis and images of MASP-3 protein in the joints of CAIA mice treated with GalNAc–MASP-3–siRNA duplex 2 compared with GalNAc–luciferase–siRNA showed lower levels of MASP-3 deposition in the knee joints synovium and forepaws synovium (Fig. 14A–I). No deposition of MASP-3 was found in the cartilage surface. Overall, the MASP-3 deposition was lower in the knee joints compared with the forepaws of mice with disease (Fig. 14E–H). Interestingly, MASP-3 deposition was also not seen in the synovial fibroblast-like cells but in different cells, and the identity of these cells is unknown at present. Notably, these MASP-3–positive cells were also present in the vicinity of fat cells known to secrete proFD (Fig. 14H). For MASP-3 IHClocalization, liverand brain from WT were used as positive controls (Fig. 14A–D), whereas liver from *MASP-1/3*^*−/−*^ mice was used as negative control (Fig. 14I). These IHC data show that MASP-3 is present in the joints, but its deposition is low, and also it decreases with GalNAc–MASP-3–siRNA duplex 2 treatment in mice with CAIA (Fig. 14E–H).

### GalNAc–MASP-3–siRNA duplex attenuated MASP-3 protein levels in the circulation of mice before and after the development of disease

**FIGURE 15.**
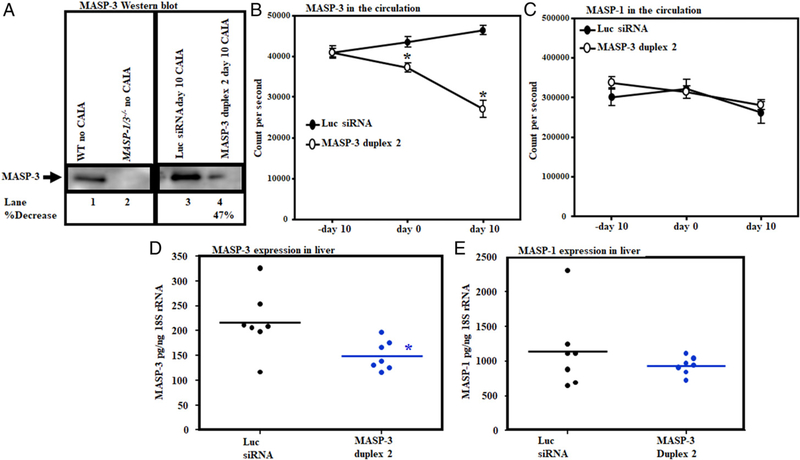
A Long-term effect of GalNAc–MASP-3–siRNA duplex 2 on MASP-1 and on MASP-3 protein in the circulation and expression in the liver before and after the development of CAIA in mice. **(A)** Western blot showing a long-term effect on the levels of MASP-3 protein at day 10 in the sera from mice with CAIA treated with GalNAc–MASP-3–siRNA duplex 2 only (lanes 3 and 4). **(B)** ELISA showing the levels of MASP-3 protein at day 0 and at day 10 in mice with CAIA injected with GalNAc–MASP-3–siRNA duplex 2 compared with the mice with CAIA injected with GalNAc–luciferase–siRNA. **(C)** ELISA showing no differences in the levels of MASP-1 protein at day 0 and at day 10 in mice with CAIA injected with GalNAc–MASP-3–siRNA duplex 2 compared with the mice with CAIA injected with luciferase siRNAs. **(D)** MASP-3 expression in the liver from mice injected with GalNAc–MASP-3–siRNA duplex 2 **(E)**. No off-target effect in the expression of MASP-1 in the liver from CAIA mice injected with GalNAc–MASP-3–siRNA duplex 2 compared with liver from CAIA mice injected with GalNAc–luciferase–siRNA. 18s RNA was used as an internal control and to calculate the expression of MASP-1 and MASP-3 in the liver for qRT-PCR. Western blot bands have been cut from the original blot to show the specific days of MASP-3 inhibition along at day 10 consistent with the liver MASP-3 expression data at day 10. Data from all mice have been included. The *p* values were calculated using t test. *p < 0.05 versus mice injected with GalNAc–luciferase–siRNAs were compared.

Sera from CAIA mice treated with GalNAc–MASP-3–siRNA or with GalNAc–luciferase–siRNA wereanalyzed at day 10forMASP-3 protein using Western blot analysis (Fig. 15A). Overall, there was a 47% decrease in the levels of MASP-3 protein, which, interestingly, was consistent with the 50% decrease in CDA as well as histopathology scores (Figs. 9A, 10A, 15A). To determine the levels of MASP-1 and MASP-3 proteins in the circulation time–resolved immunofluorometric assay was used as described in Materials and Methods (Fig. 15B, C). There was a significant (*p* = 0.033) decrease of 14.4% in the levels of MASP-3 at day 0 in mice injected with GalNAc–MASP-3–siRNA duplex 2 compared with mice injected with GalNAc–luciferase–siRNA (Fig. 15B). At day 10, there was a significant (p = 0.011) decrease of 42% in the levels of MASP-3 in mice injected with GalNAc–MASP-3–siRNA duplex 2 compared with mice injected with luciferase siRNAs (Fig. 15B). No significant differences were seen in the levels of MASP-1 at any time point before and after injections and also before and after the induction disease (Fig. 15C). The levels of MASP-2 and MAp44 were not examined because of the limited amount of serum from each mouse available for these studies. These data show that there was an effect on the levels of MASP-3 without substantial off-target effects on MASP-1 in mice injected with GalNAc–MASP-3–siRNA duplex 2 or GalNAc–luciferase–siRNA (Fig. 15A–C).

### GalNAc–MASP-3–siRNA duplex decreased the expression of MASP-3 in the liver of mice with CAIA

Upon sacrifice, livers from the above CAIA mice were snap frozen. Total RNA was extracted to examine the expression of MASP-1 and MASP-3 from mice treated with GalNAc–MASP-3–siRNA duplex 2 or GalNAc–luciferase–siRNA (Fig. 15D, 15E). There was a significant (*p* = 0.025) decrease in the expression of MASP-3 in the liver of mice with CAIA treated with GalNAc–MASP-3–siRNA duplex 2 compared with the mice with CAIA treated with GalNAc–luciferase–siRNA (Fig. 15D). Overall, there was a 31% decrease in the MASP-3 expression in the liver of CAIA mice treated with GalNAc–MASP-3–siRNA duplex 2 compared with the mice with CAIA treated with GalNAc–luciferase–siRNA. No significant change (*p* = 0.35) was seen for MASP-1 mRNA abundance in the liver from CAIA mice treated with GalNAc–MASP-3–siRNA compared with the mice with CAIA treated with GalNAc–luciferase–siRNA (Fig. 15E). These data show that GalNAc–MASP-3–siRNA duplex 2 decreased the expression of MASP-3 and not of MASP-1 in the liver.

## DISCUSSION

MASP proteins (MASP-1 and MASP-2) were initially identified through their interaction with MBL (49–51). MASP-3 was identified shortly thereafter, also through interactions with MBL (23, 25). MASP-1 and MASP-3 are alternatively splice forms derived from the *MASP1/3* gene along with the additional splice variant MAp44, whereas MASP-2 is encoded by the separate MASP2 gene. Although MASP-1 and MASP-3 are derived from the same gene, their functions are quite different. MASP-1 has recently been shown to activate MASP-2 via a cleavage event, thus enabling the MASP-2–mediatedcleavage of C2 and C4 and the construction of the C3 convertase C4b2a for the LP (52, 53). MASP-1 is also suggested to be responsible for the activation of MASP-3, which, in contrast to MASP-2, is responsible for the cleavage of proFD to active FD for the AP (54). Thus MASP-3 is an important regulator of the AP, whereas MASP-1 is a more general MASP activator. Gene deletion experiments that generated *MASP1*/3^−/−^ mice resulted in both inactivated MASP-1 and MASP-3 functions, rendering our understanding of the individual roles of these two enzymes uncertain. Specific deficiency of MASP-3 is rare but has been observed in humans. Mutations in *MASP1* exon 12 render MASP-3 inactivated and are responsible for the development of Malpuech–Michels–Mingarelli–Carnevale syndrome (55–57). This indicates that MASP-3 plays a developmental role as well as its role in the activation of the AP of complement.

In our studies of the role of MASPs in an experimental RA, we have previously shown that disruption of the *MASP1* gene, resulting in the loss of both MASP-1 and MASP-3, almost completely blocked disease in the CAIA model (14). Subsequently, we found that overexpression of MAp44, a competitive inhibitor of MASP-1, MASP-2, and MASP-3, nearly completely blocked the development of disease (5). Dissociation of the effects of MASP-1 and MASP-3 genetically was technically challenging, however, requiring the selective disruption of exons unique to MASP-3.

Progressin thedesign ofsiRNAs, however, afforded us asecond option, namely, to design siRNAs that could distinguish between MASP-1 and MASP-3 mRNAs. We approached this in two ways. Working with the functional genomics core of the University of Colorado, we designed shRNA sequences that could be expressed by lentiviral infection. The lentiviral construct was successful at targeting human MASP-3 mRNA but not MASP-1 or MASP-2. HepG2 and T98G cells chosen for both of these are high MASP-3–expressing cell lines (44). We saw no significant change in MASP-1 transcripts, whereas MASP-3 transcripts were almost entirely absent after lentiviral infection in T98G cells (Fig. 2C). Furthermore, the effect was durable and lasting for months in T98G cells (Supplemental Fig. 2A). In HepG2 cells, no effect was seen on MASP-1 and MASP-2, whereas there was a significant decrease in MASP-3 (Fig. 2F). High levels of MASPs in mouse liver compared with human liver indicate species-specific differences.

To evaluate specific silencing of MASP3 in vivo and mouse CAIA model, we used Alnylam Pharmaceuticals’ liver-specific GalNAc-siRNA delivery technology. The triantennary GalNAc ligand engages the hepatocyte-specific ASGPR receptor enabling siRNA uptake. siRNAs conjugated to a GalNAc have been used extensively to silence liver-expressed genes in both preclinical species and in the clinic. This platform has demonstrated robust clinical translation across multiple liver target genes (42). For example, Inclisiran (ALN-PCSsc) significantly reduced the levels of PCSK9, a target for low-density lipoprotein (LDL) cholesterol, for at least 6 mo (43). In contrast, lentiviral constructs are rapidly cleared when administered systemically (58), making efficient knockdown of a target by this means difficult. Multiple GalNAcsiRNA conjugates specific for mouse MASP3 were designed and evaluated in in vitro studies and healthy mice to select a duplex for testing in the CAIA model.

The MASP-3 duplex chosen for our in vivo studies decreased liver MASP-3 mRNA by ~50% 7 d after a single dose (Fig. 6). In our first CAIA study, however, providing three siRNA doses on days −5, 0, and +3 (relative to inductionof disease)resulted in an almost complete elimination of MASP-3 mRNA (Fig. 7D). MASP-3 protein in the serum had decreased by 50% (Fig. 6A). This may be due, in part, to other tissue sources for MASP-3 and to the half-life of MASP-3 protein in the serum. CAIA disease progression was attenuated by the GalNAc–MASP-3–siRNA treatment in mice but not completely blocked. Histopathology scores for inflammation, pannus, cartilage damage, and bone damage were also consistent with the CDA. C3 deposition in the knee joints were also decreased and consistent with the decrease in MASP-3 deposition in the synovium of knee joints and forepaws. The attenuation of disease is consistent with the diminished presence of serum MASP-3 (Fig. 15B).

We have shown that CAIA is dependent on the AP as *FD*^*−/−*^,*MBL A/C/FD*^*−/−*^, *C1q*^*−/−*^/*FD*^*−/−*^, and *MASP-1/3*^*−/−*^ mice are resistant to CAIA (14, 59). An essential step in the AP is the cleavage of proFD to FD. Several groups have shown, using in vitro methods, that MASP-3 appears to be the essential enzyme that activates FD. One group has generated inhibitors specific for MASP-1, 2, and 3, and using these inhibitorshas shown that MASP-3 alone appears to be responsible for proDF cleavage (31). Using various sera depleted for MASP-3 to varying extents compromises the conversion of proFD to DF (32). We show, in vivo, cleavage of proFD to DF and reconstitution of the AP activation in *MASP-1/3*^*−/−*^ mice by active rMASP-3 (Fig. 4). This demonstrates that MASP-3 is sufficient to reconstitute the AP in vivo but does not yet demonstrate that it is necessary. As MASP-1 and MASP-3 are generated by alternative splicing of a single gene, mice in which one or the other splice forms are genetically disrupted will be needed to formally prove this hypothesis in vivo.

Examining the time course of disease development in GalNAc–MASP-3 siRNA-treated mice, we note that disease is markedly inhibited through day 6 (69%) (Fig. 9). Then, a rapid acceleration of disease occurs on day 7 leading to the final inhibition of disease to 50% of control (Fig. 9A). We postulate that a mini breakthrough of MASP-3 expression may have occurred at this point. Alternatively, one can speculate that there is a possibility that an extrahepatic MASP-3 contribution by brain or inflammatory cells could have played a role in the breakthrough. Although a blood–brain barrier might exist, during acute inflammatory conditions, there could be a mini breakthrough in this blood–brain barrier. Although technically infeasible in this study, a future study will be designed to examine serum daily for MASP-3 levels to test this hypothesis.

Importantly, GalNAc–MASP-1–siRNA had no significant effect on CAIA even after inhibiting more than 95% of MASP-1 expression in the liver as well as MASP-1 protein in the circulation (data not shown), suggesting that the contribution of the *MASP1/3* gene to CAIA is primarily through MASP-3 but not through MASP-1, although both are predominately generated by the liver. Given the suggested role of MASP-1 as an activator of MASP-3, this result demonstrates the existence of other activators of MASP-3. We also examined the effect of GalNAc–MASP-2 siRNA (data not shown) and obtained results similar to those we obtained with *MASP-2*^*−/−*^ mice (34). Given that the liver is also the major source of MASP-2, this result was expected. Interestingly, it has been shown using paired samples that high levels of MASP-3 versus MASP-2 are present in the synovial fluid as well as in the plasma of RA patients (17). Studies combining siRNAs targeting combinations of MASPs are planned for the future.

A limitation of our study is that liver-targeted MASP-3–siRNA duplexes did not completely inhibit MASP-3 expression as we observed MASP-3 protein in the circulation of mice after siRNA treatment. This may be due to the contribution of other tissues during acute inflammatory conditions or alternately may be due to the inherent stability of MASP-3 protein. Indeed, although we know that transcripts from the *MASP1* gene derive from other tissues besides liver (44), we do not yet know the tissue specificity of the splicing event. Furthermore, we have no information on the rate of turnover of MASP-3 during steady state or during inflammation. All of these parameters will affect our interpretation of the data presented in this study. Thus, the partial inhibition of disease may simplybe due to the partial removal ofserum MASP-3. This question can only be answered by the construction of a MASP-3 exon tissue-specific knockout mouse strategy, allowing removal of all MASP-3 or tissue-specific expression while leaving MASP-1 and MAp44 intact.

The liver is seldom thought of as an organ associated with joint disease in RA. Given its role as a major source of soluble complement components (10, 11, 34), we suggest that this role should be reconsidered. Recently, it has been proposed that the etiology of RA involves a combination of genetics and epigenetics leading to an increased likelihood of developing an immune response to posttranslationally altered self (8). Deposition of Ab within the synovial space leads to the initiation of a joint specific immune response. This, however, is not sufficient. For the response to become self-sustaining, we propose that a second hit of liver-specific secretion of complement components necessary for the AP is necessary. Our treatment schedule (injections on days −5, 0, and +3 or on day −10, day −5, or day 0) has focused on the role of complement in the early phase of RA disease development. It will be important to employ our GalNAc–MASP-3–siRNA therapeutics in established disease to test whether complement remains a driver at all times or just at early times.

Given the importance of complement for innate immunity, it is likely that a therapeutic that strongly inhibits this system may increase susceptibility for infections. In this regard, it is, perhaps, beneficial that the GalNAc–MASP-3–siRNA only partially blocks MASP-3 protein levels. Interestingly, a recent study found that relatively small amounts of FD are sufficient to activate the AP in mice (60). Because MASP-3 deposition was low in the joints, we speculate that a small amount of MASP-3 can activate the AP locally or systemically. This idea is supported by the fact that *MASP-1/3*^*+/−*^mice, which have suboptimal levels of MASP-3, were equally susceptible to CAIA when comparing with the WT mice (data not shown).

In conclusion, this represents the first study, to our knowledge, in vivo demonstrating an important role of MASP-3 (separate from MASP-1) in the development of RA. It supports the contention that the liver plays an important role in RA and supplies a candidate enabling the second hit to drive disease. Finally, it provides a further demonstration of the therapeutic potential for novel GalNAc–MASP-3–siRNA duplexes.

## Supplementary Material

1

## Abbreviations used in this article:

AJM: all joint mean
AP: alternative pathway
ASGPR: asialoglycoprotein receptor
CAIA: collagen Ab–induced arthritis
CDA: clinical disease activity
FD: factor D
GalNAc: N-acetylgalactosamine
hu shMASP-3: human shMASP-3
IHC: immunohistochemical
LP: lectin pathway
MASP: MBL-associated serine protease
MBL: mannan-binding lectin
PCSK9: proprotein convertase subtilisin-kexin type 9
PRM: pattern recognition molecule
proFD: profactor D
PVDF: polyvinylidene difluoride
qRT-PCR: quantitative RT-PCR
RA: rheumatoid arthritis
RNAi: RNA interference
rM3: recombinant MASP-3
rM3 S/A: inactive or mutated rM3
sh: short hairpin
siRNA: small interfering RNA
T blue: toluidine blue
WT: wild type
