# Supplementary material for: Targeting of Liver Mannan-Binding Lectin–Associated Serine Protease-3 with RNA Interference Ameliorates Disease in a Mouse Model of Rheumatoid Arthritis
Source: Immunohorizons. Author manuscript; Available in PMC 2018 Nov 7. (PMC6220895; doi:10.4049/immunohorizons.1800053)
Supplement: 1 [file NIHMS990435-supplement-1.pdf]

A. Plasmid-GFP-No shScramble at 24 hrs

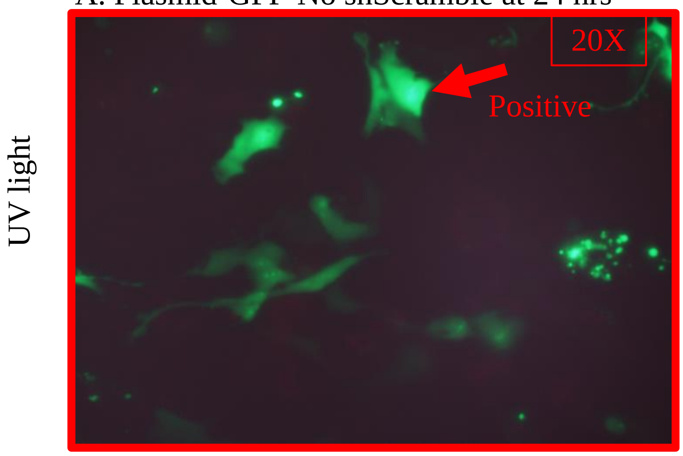

C. Plasmid-GFP-No shScramble at day 15

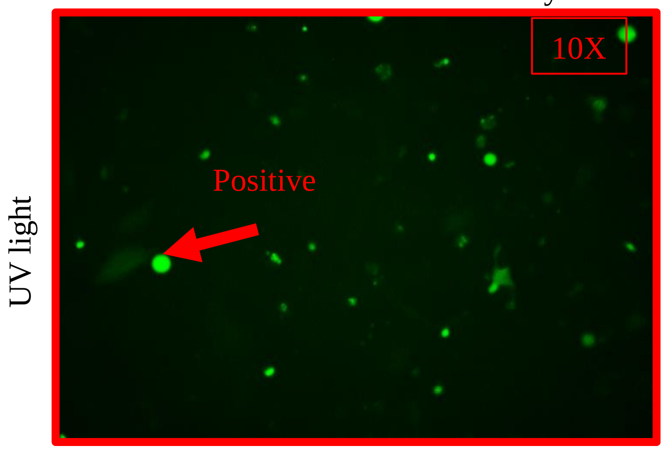

Supplement S1 Assessment of the transfection efficiency in T98G cells using GFP expressing plasmid. TG98 cell were transduced with GFP-expressing plasmid in parallel in a 6-well plate with each experiment and examined under light microscope and UV light. **A**. GFP (Green color) expressing T98G cells under UV light at 24 hours (magnification at 20X). **B.** T98G cells in culture at day 15 under UV light showing GFP-expressing green cells (magnification at 10X).

Supplement S2

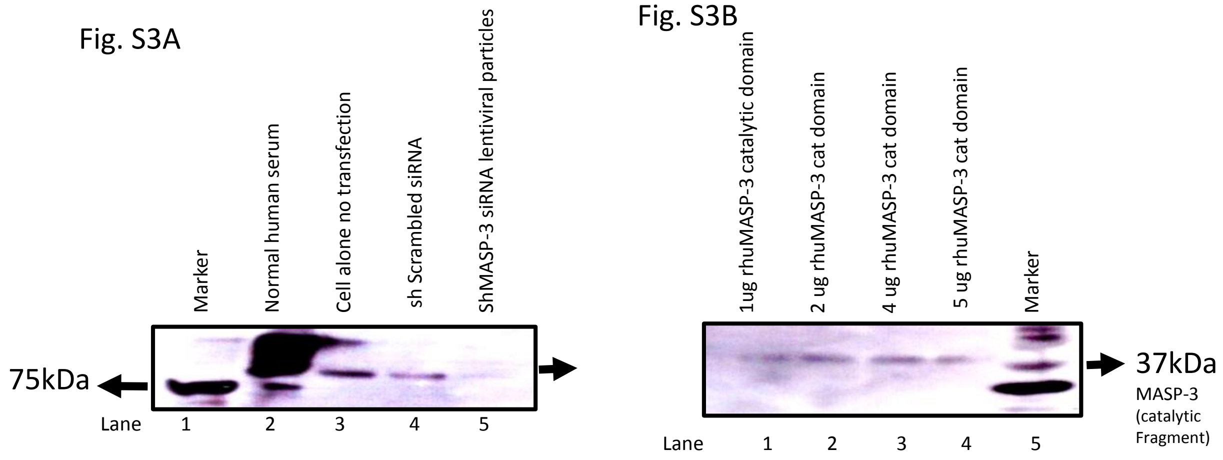

Supplement S2 Long-term Western blot analysis showing a decreased levels of human MASP-3 protein after transfection with hu shMASP-3 RNA or shscramble RNA in the supernatants of cultured T98G cells. The supernatants were collected, at day 60 and concentrated before using for Western blot analysis. Human MASP-3 protein was immunoprecipated using mannan-agarose beads before analyzing using a 10% SDS-PAGE as mentioned in the Materials and Methods. Normal human serum (NHS) was used as a positive control to identify the specific band of MASP-3 in the supernatants. The concentrated supernatants were immuneprecipatated and analyzed using Western blot analysis. **Fig S2A.** Standard protein marker (lane 1), NHS (lane 2), supernatant from cells alone without any transfection (lane 3), supernatants from cells transfected with shScramble RNA as a control (lane 4), supernatants from cells transfected with shMASP-3 RNA (lane 5). **Fig S2B.** Western blot analysis showing the various concentration of rMASP-3 protein ranging from 1ug to 5ug (lanes 1-4) and standard protein marker (lane 5). An equal amounts of the concentrated supernatants were loaded in each well.

Supplement S3

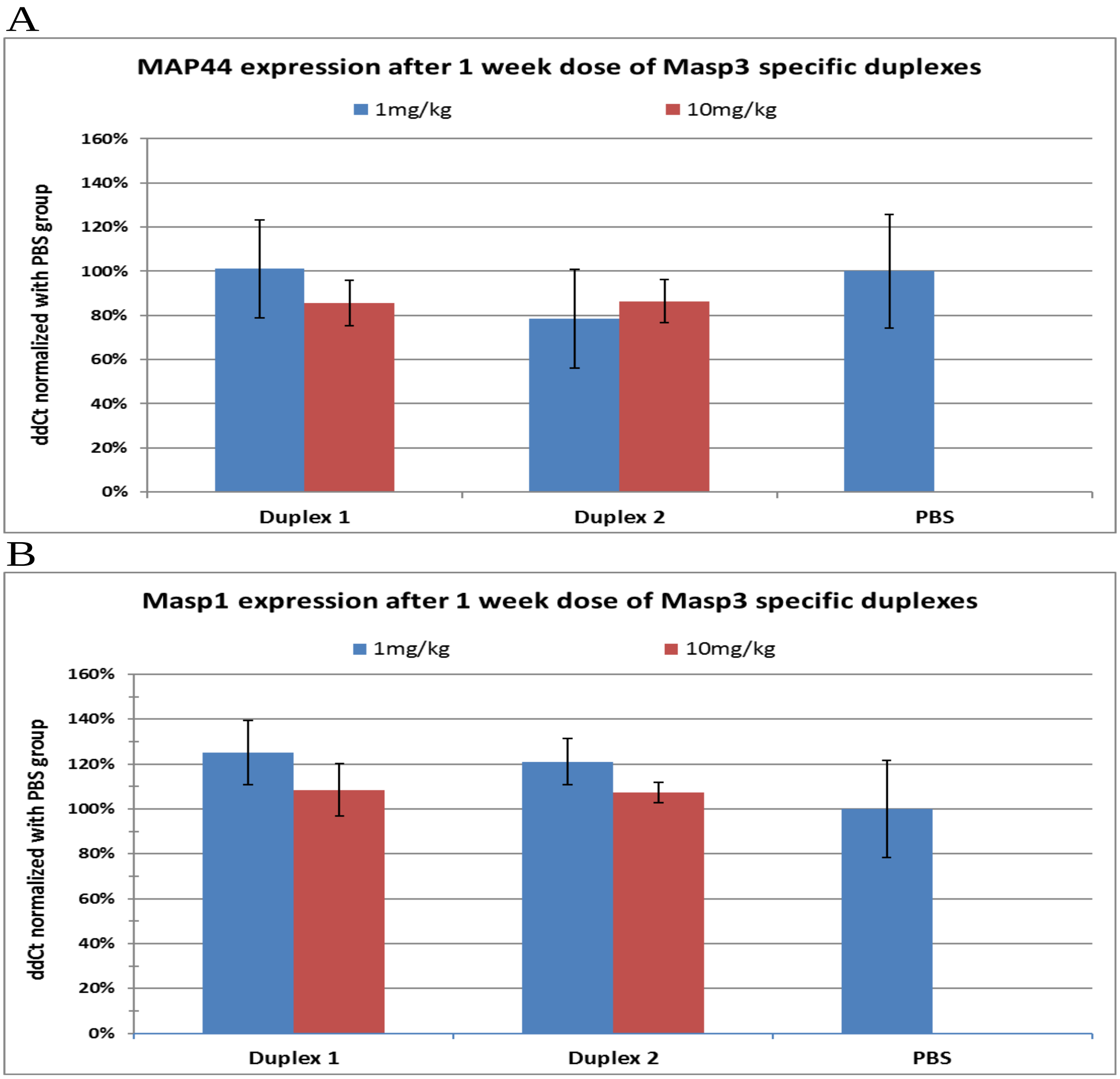

Supplement S3 Quantitative RT-PCR analysis of MAP44 expression and MASP1 expression from the liver of WT mice injected with GalNAc-MASP-3-siRNA duplexes. **S3A**. MAp44 expression. **S3B.** MASP-1 expression. C57Bl/6J mice were subcutaneously injected with two different doses of GalNAc-MASP3-siRNA duplexes – 1mg/kg (blue) and 10mg/kg (red). Liver was collected from mice on day 7 after injection. Gene expression was examined using qRT-PCR. Group of mice treated with PBS is used for normalizing the gene expression (ddCt).

GAPDH was used as an internal control to calculate the gene expression. N = 3 in each group of duplex (#1 and #2) total n = 15.
